# Synthesis and in silico studies of new thiophene-isoquinolinone hybrids as potential larvicides against *Culex pipiens*

**DOI:** 10.1038/s41598-025-13063-7

**Published:** 2025-07-31

**Authors:** Mahmoud Kamal, Mohamed H. Hekal, Fatma S. M. Abu El-Azm, Eslam M. Hosni, Yasmeen M. Ali, Abdullah Yahya Abdullah Alzahrani, El-Hady Rafat

**Affiliations:** 1 Department of Entomology, Faculty of Science, Ain Shams University, Abbassia, Cairo, 11566 Egypt; 2 Department of Chemistry, Faculty of Science, Ain Shams University, Abbassia, Cairo, 11566 Egypt; 3 Department of Chemistry, Faculty of Science, King Khalid University, Abha, Saudi Arabia

**Keywords:** Thiophene-isoquinolinone hybrids, *Culex pipiens*, Larvicidal activity, Acetylcholinesterase (AChE), Nicotinic acetylcholine receptor (nAChR), Molecular docking, Density functional theory (DFT), Insecticide resistance, SAR, Neurotoxic insecticides, Organic chemistry, Computational chemistry, Enzymes

## Abstract

Mosquito-borne diseases remain a significant global public health challenge. This challenge is further exacerbated by the growing resistance of disease-vector species such as *Culex pipiens* to conventional insecticides. In this study, we present the design, synthesis, and biological assessment of a new series of thiophene-isoquinolinone hybrids as potential larvicides. Among the synthesized compounds, derivatives 5f, 6, and 7 showed significant larvicidal effectiveness against *Culex pipiens* larvae, with LC₅₀ values of 0.3, 0.1, and 1.85 µg/mL, respectively. Notably, all twelve thiophene-isoquinolinone derivatives were much more toxic than the reference organophosphate insecticide chlorpyrifos (LC₅₀ = 293.8 µg/mL), demonstrating the strength of these chemical structures. Interestingly, the synthetic intermediate compound 1a, a thiophene-based half-ester, exhibited the highest activity (LC₅₀ = 0.004 µg/mL), outperforming all final derivatives despite not being fully optimized. Mechanistic bioassays showed consistent neurotoxic symptoms that suggest a disruption of cholinergic function. Molecular docking and molecular dynamics simulations supported this observation, revealing strong and specific interactions with both acetylcholinesterase (AChE) and nicotinic acetylcholine receptors (nAChRs), which points to a possible dual-target mechanism. Density Functional Theory (DFT) calculations further confirmed the favorable electronic properties and reactivity of the active compounds. The structural variety within this series, along with consistently high potency, may lower the risk of cross-resistance and aid resistance management strategies through compound rotation or combination. Overall, these findings highlight thiophene-isoquinolinone hybrids as a promising option for developing next-generation larvicides that target neurophysiological pathways in insect vectors.

**Supplementary Information:**

The online version contains supplementary material available at 10.1038/s41598-025-13063-7.

## Introduction

Mosquitoes are among the most effective vectors of infectious diseases, transmitting a wide range of harmful pathogens that present major risks to global public health. Species like *Culex pipiens*, *Aedes aegypti*, and *Anopheles gambiae* are particularly notorious for spreading viruses, bacteria, and parasites, resulting in millions of infections and significant mortality each year^1–4^. For example, *Culex pipiens* is a key transmitter of arboviruses such as West Nile virus and St. Louis encephalitis virus, as well as parasitic diseases like avian malaria^3^. Recent research has also uncovered its role in harboring and transmitting harmful bacteria, including *Bacillus cereus* and *Staphylococcus warneri*, which can contaminate food supplies and compound public health issues^2^. Mosquitoes’ ability to adapt, survive, and develop resistance to control methods makes them even more difficult to manage and keeps them as ongoing threats.

Chemical insecticides have been a key part of mosquito control, particularly during outbreaks of diseases spread by vectors. Different classes of insecticides, like pyrethroids, organophosphates, and carbamates, have been used widely to lower mosquito numbers and reduce the spread of disease^5–10^. However, the extensive and prolonged use of these chemicals has raised significant environmental and public health concerns, including ecosystem damage, harmful effects on non-target species, and the rapid development of insecticide resistance within mosquito populations^11–14^. This resistance has greatly diminished the efficacy of many traditional insecticides, highlighting the urgent need for innovative chemical solutions with new mechanisms of action to effectively combat these evolving threats^11–14^. In response to these important challenges, researchers are turning to alternative strategies like biological control, genetic modification, and integrated vector management (IVM). These methods show promise for sustainable, long-term mosquito control. However, chemical methods are still crucial for quick action during epidemics and emergencies.

Adding to these problems, the effects of climate change are increasing the risks related to mosquito-borne diseases^4,15^. Rising temperatures and shifting weather patterns are expanding the habitats of mosquitoes, creating more favorable conditions for their survival and reproduction. As a result, regions that were once free from diseases like West Nile virus and avian malaria are now becoming more susceptible to outbreaks^4,16–18^. This active interaction between climate change, mosquito behavior, and disease spread shows the need for practical strategies to tackle the rising threat of diseases carried by mosquitoes. A well-rounded approach that includes new chemical solutions, sustainable control methods, and climate-responsive policies is crucial to reduce the increasing health risks from mosquitoes in a warming world.

Heterocyclic compounds play a pivotal role in therapeutic chemistry due to their broad pharmacological potential and structural versatility^19–23^. Among them, thiophene, a five-membered sulfur-containing ring, has become an important pharmacophore in medicinal chemistry due to its favorable electronic properties and its ability to improve biological activity. The US FDA has approved seven small-molecule drugs that contain the thiophene moiety, making it the fourth most approved heterocycle recently^24^. This moiety is present in numerous therapeutically important agents such as penthiopyrad (antifungal)^25^, morantel (anthelmintic)^26^, cefoxitin (antimicrobial)^27^, raltitrexed (anticancer)^28^. Notably, several thiophene-containing compounds have also demonstrated acetylcholinesterase (AChE) inhibitory activity^29^, suggesting their potential as neurotoxic agents for pest control. Since AChE is a key enzyme responsible for terminating synaptic transmission in insect nervous systems, thiophene-based inhibitors may mimic the mode of action of conventional insecticides such as organophosphates and carbamates.

Isoquinoline alkaloids are significant *N*-based heterocyclic compounds that can be found in abundance throughout the plant kingdom, such as *Amaryllidaceae*, *Rubiaceae*, *Magnoliaeace*, *Papaveraceae*, *Berberidaceae*, *Menispermaceae*, and other plant families^30^. Foregoing studies have verified that isoquinoline alkaloids have a variety of biological activities and structural frameworks, including insecticidal, antidiabetic antitumor, antifungal, anti-inflammatory, antibacterial, antiparasitic, antioxidant, antiviral, and neuroprotective^31,32^.

**Fig. 1:**
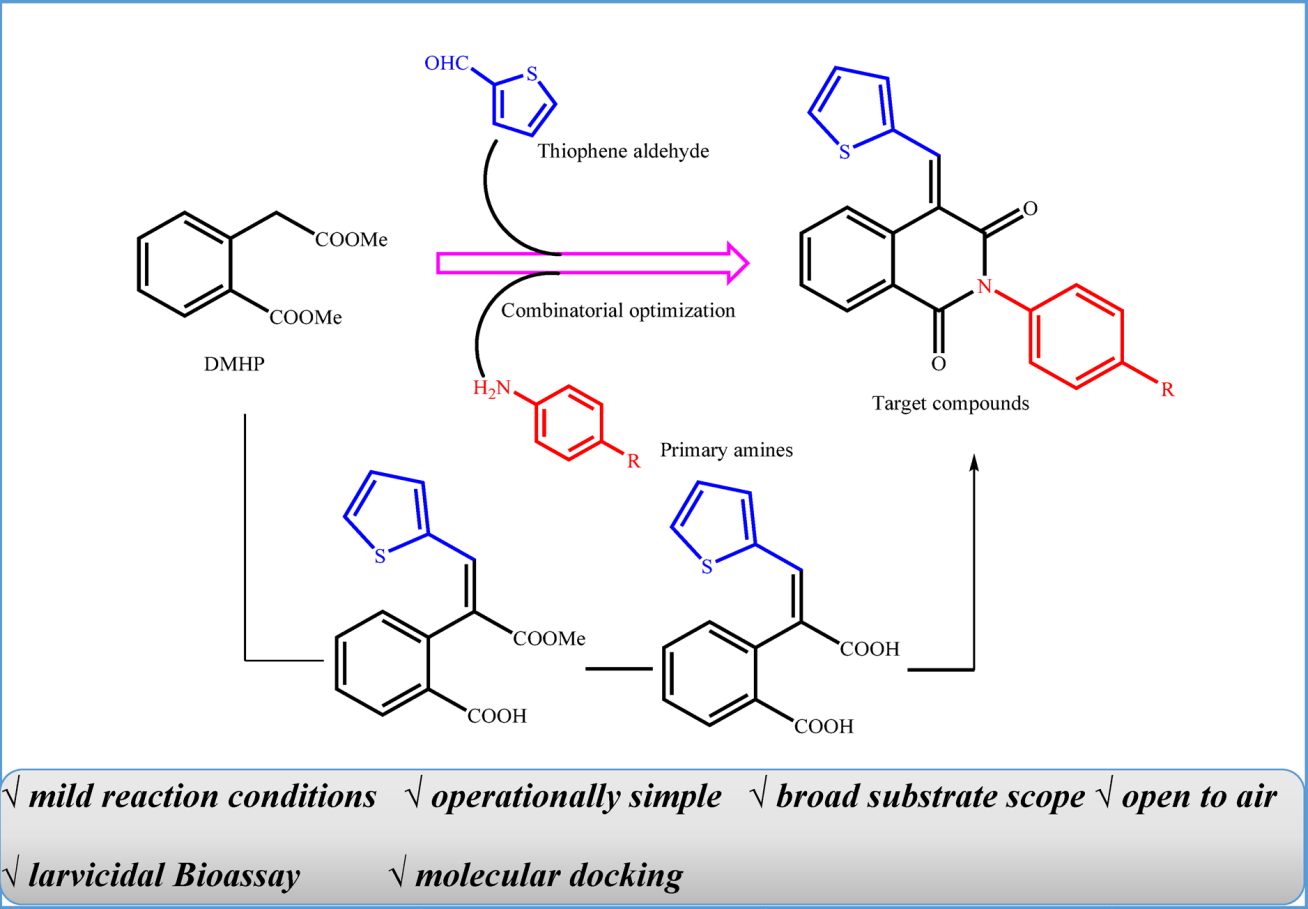
The design strategy of the target compounds.

The integration of thiophene and isoquinoline substructures holds significant potential for the discovery of novel insecticidal agents with enhanced biological activity, as each moiety contributes complementary properties—thiophene for its electronic characteristics and AChE inhibitory activity, and isoquinoline for its diverse biological effects—potentially enabling synergistic interaction with multiple neural targets such as AChE and nAChR. Building on this concept, this study focuses on the design, synthesis, and evaluation of a new series of thiophene-isoquinolinone hybrids as potential larvicidal agents against *Culex pipiens* mosquitoes (Fig. 1). To achieve this, we employ a combination of experimental and computational approaches, including molecular docking, molecular dynamics simulations and density functional theory (DFT) calculations, to assess the biological efficacy and chemical properties of the synthesized compounds. The specific objectives of this study are: (1) to design and synthesize novel thiophene-isoquinolinone derivatives using efficient synthetic strategies, (2) to evaluate their larvicidal activity against *Culex pipiens*, (3) to investigate their mechanisms of action through molecular docking and molecular dynamics simulations, and (4) to analyze their electronic and structural properties using computational chemistry techniques. Ultimately, this research aims to advance the development of next-generation insecticidal agents with improved efficacy and selectivity, addressing the growing challenge of insecticide resistance and contributing to more effective mosquito control strategies.

## Results and discussion

### Chemistry

**Fig. 2:**
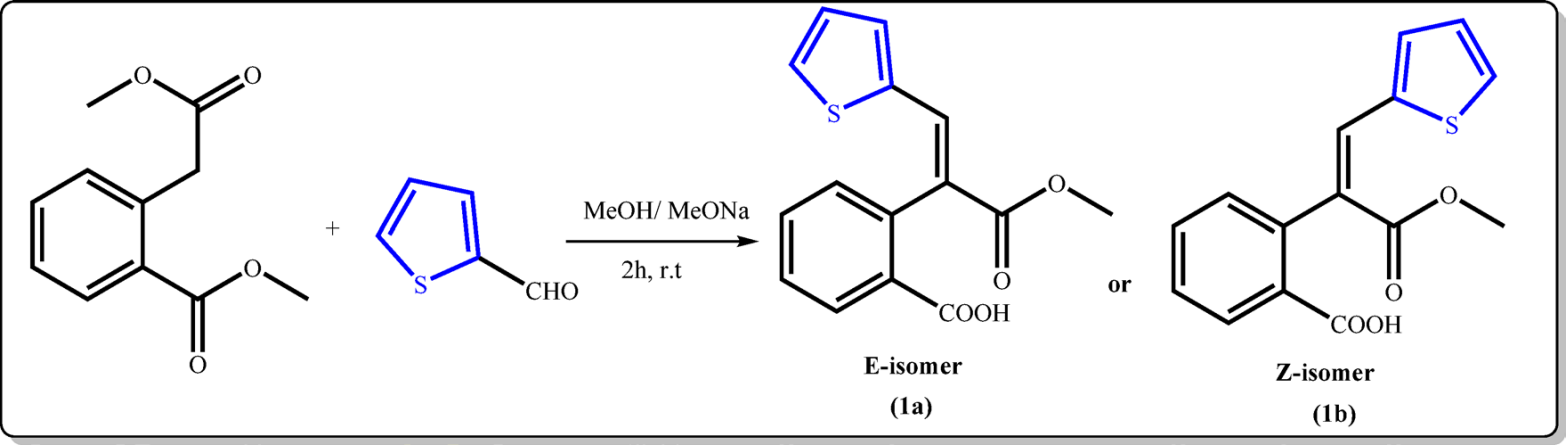
Synthesis of the half ester 1.

As a part of our dedication to develop new heterocyclic compounds and assessing their potential in medicinal and biological applications^33–51^, we synthesized a new series of *N*-substituted isoquinolinone derivatives and evaluated their insecticidal activity. The key starting material was synthesized *via* Stobbe-type condensation reaction^52–54^ between dimethyl homophthalate and thiophene-2-carbaldehyde, using sodium methoxide in dry methanol, resulting in the half ester **1** (*E*- or *Z*-isomer) as shown in Fig. 2.

**Fig. 3:**
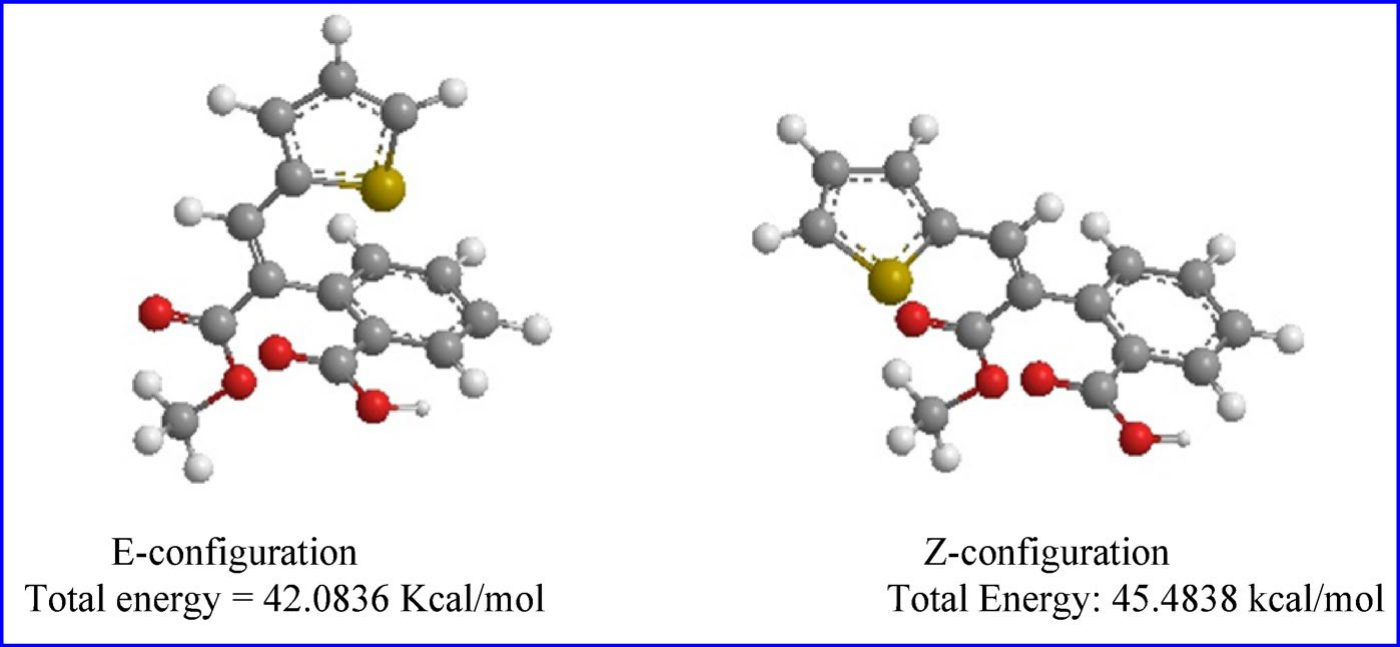
Space models of the *E-* and *Z*-configurations.

The preferential formation of the *E*-isomer **1a** may be interpreted by considering the stability of the final product. By constructing space models for these isomers through PerkinElmer ChemDraw Professional 16.0, it was found that *E*-configuration is thermodynamically more stable than *Z*-configuration according to MMFF94 calculations. The total energy of the *E*-isomer is 42.0836 Kcal/mol, while that of the *Z*-isomer is 45.4838 Kcal/mol, which is 3.40 Kcal/mol larger than that of the *E*-isomer as shown in Fig. 3.

**Fig. 4:**
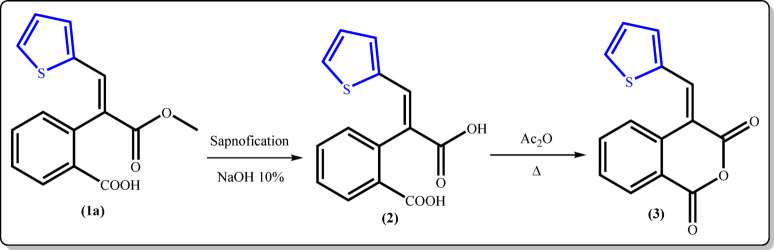
Synthesis of isochroman-1,3-dione derivative 3.

Mild saponification of the half ester **1a** gave the *E*-dibasic acid **2**, which subsequently underwent cyclization using freshly distilled acetic anhydride to afford isochromane-1,3-dione **3** as the sole product in good yield (Fig. 4). The structure of the anhydride **3** was substantiated from spectral data besides the correct elemental analysis.

**Fig. 5:**
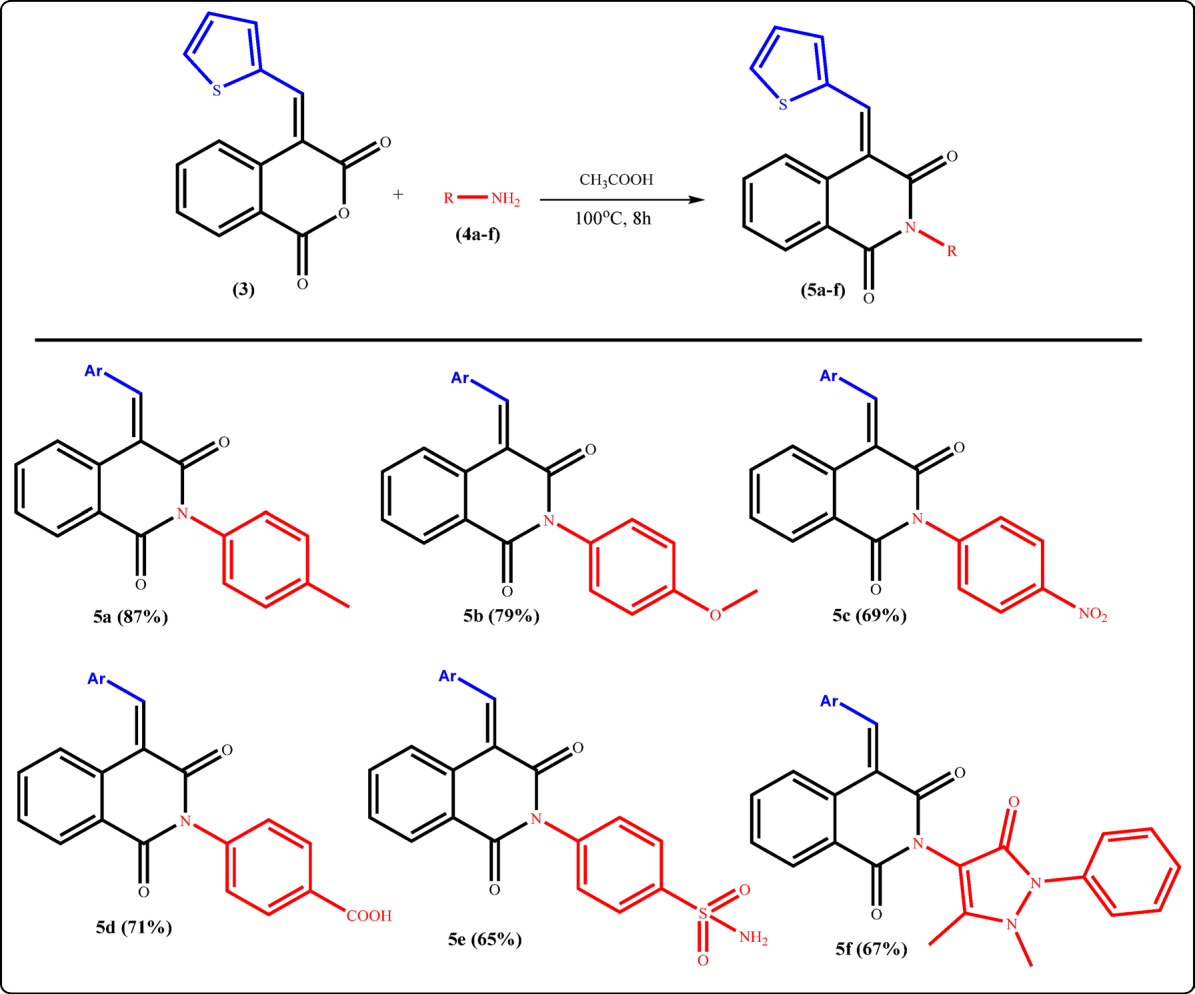
Reaction of isochroman-1,3-dione 3 with various aromatic amines.

The functionality in isochroman-1,3-dione **3** renders it a valuable precursor for preparation of new isoquinolinones bearing a thiophene moiety. Thus, the proclivity of the isochromene **3** towards various nitrogen nucleophiles such as ammonium acetate, primary amines, and some acid hydrazides has been investigated. Initially, we examined the reaction between isochroman-1,3-dione **3** and *p*-toulidine **4a** as a *N*-nucleophile source, which afforded isoquinolinone **5a** (**Supplementary Data**). After screening of conditions, we finally found that the reaction of **3** (1.0 equiv) with the amine (2.0 equiv) in the presence of glacial acetic acid at 100 °C for 8 h produced **5a** in excellent yield. With the conditions in hand, new series of isoquinolinone derivatives were synthesized by nucleophilic displacement reaction on isochroman-1,3-dione **3** by substituted primary amines. Subsequently, the suitability of different aromatic amines for this transformation was examined (Fig. 5).

A variety of aryl groups containing electron-donating or electron-withdrawing substituents successfully furnished the desired isoquinoline-1,3-dione-based derivatives. For instance, treatment of **3** with *p*-anisidine, *p*-nitro aniline, *p*-amino benzoic acid, and/or sulfanilamide afforded the expected cyclized products **5b-e**, respectively, in moderate to good yields. The structures of **5a-e** were evidenced by spectral data besides the elemental analysis. For example, ^1^H-NMR spectrum of compound **5a** showed a singlet signal at 2.39 ppm which was attributed to protons of CH_3_ group, as well as the absence of vibrational coupling of carbonyl groups of **3**, as observed in its IR spectrum, further confirmed the assigned structure.

On the other hand, refluxing **3** with heterocyclic amine such as 4-amino antipyrine provided the corresponding thiophene-based product **5f** as a sole product in fairly good yield as illustrated in (Fig. 5).

**Fig. 6:**
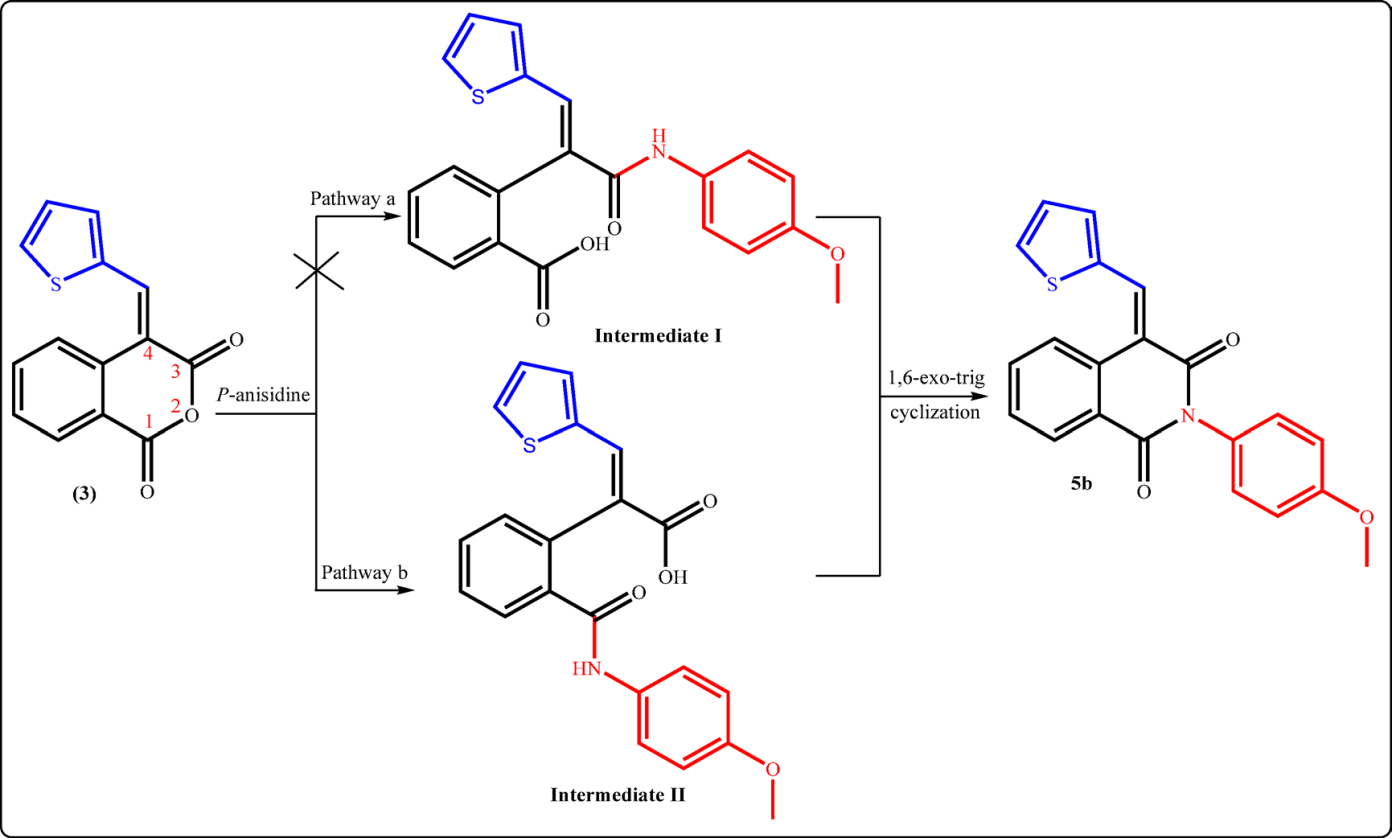
Mechanistic pathways for the formation of compound 5b.

The plausible mechanism for treatment of the anhydride **3** with primary amines (for example, *p*-anisidine) could be visualized in Fig. 6. The first step involved the nucleophilic attack by the lone pair of electrons of NH_2_ group at the carbonyl–carbon atom of isochromanone, proceeding through one of two potential mechanistic pathways (a or b) leading to the formation of intermediates (I or II), followed by 6-exo-trig cyclization to achieve the desired product.

The preferential formation of the desired product **5b** through the mechanistic pathway (b) can be inferred from computational chemical investigations. It was found that the charge density on C_1_-isochromanone is + 0.593, while on C_3_-position is + 0.591, which confirmed the relatively higher electron-deficiency of C_1_- than that of C_3_-position. As a result, C_1_-site has a slightly higher chance of being attacked by the nucleophilic reagents (pathway b).

**Fig. 7:**
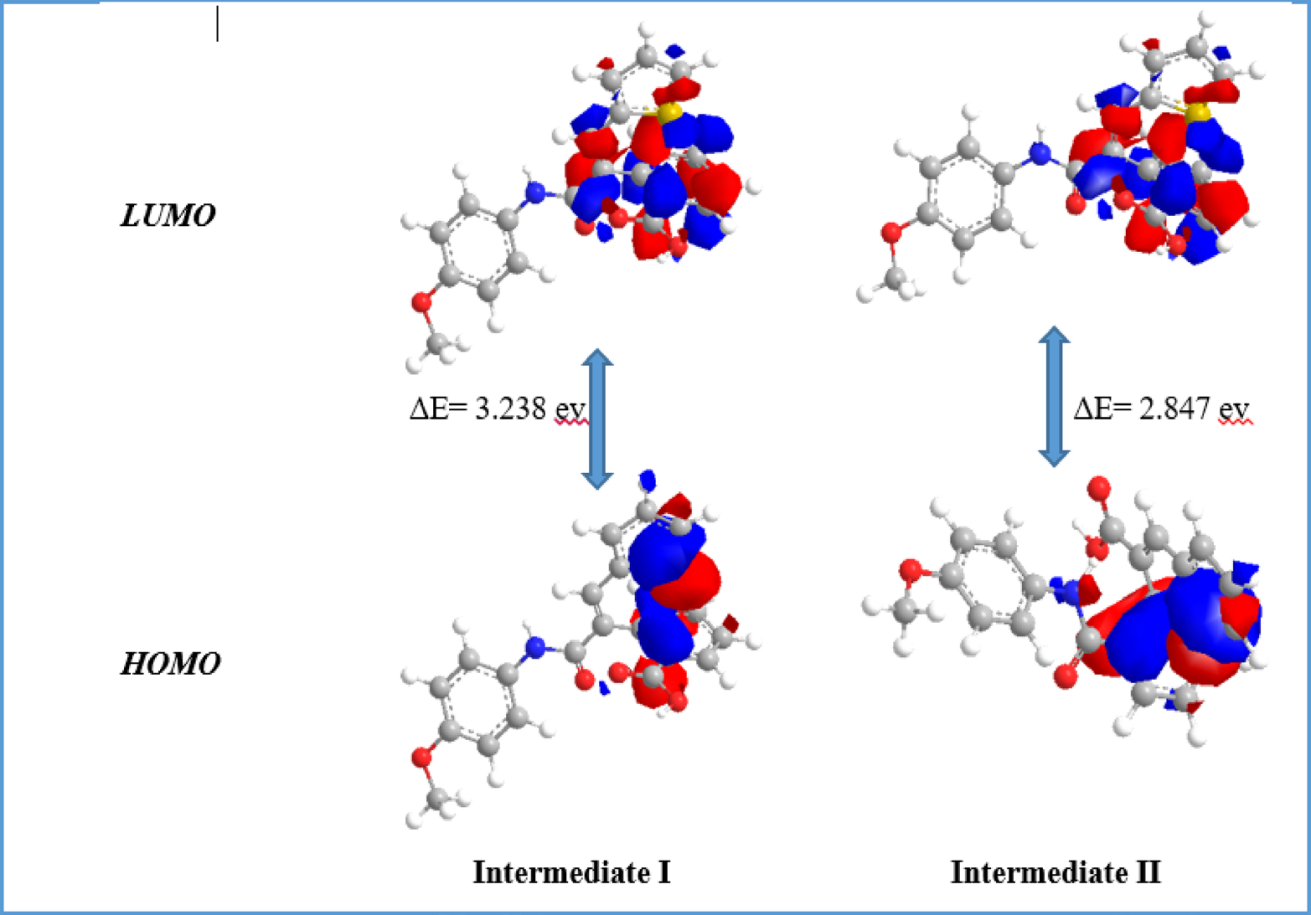
The HOMO–LUMO energy gap of the intermediates I and II.

The frontier molecular orbital perspective (FMO) was the basis for supporting the previous findings. The HOMO-LUMO energy gap (Δ*E*) for the intermediate **I** is 3.28 eV, while for **II** is 2.84 eV, which is 0.44 eV lower than that of **I** as presented in Fig. 7. Subsequently, the formation of the intermediate **II** is favored over **I**.

**Fig. 8:**
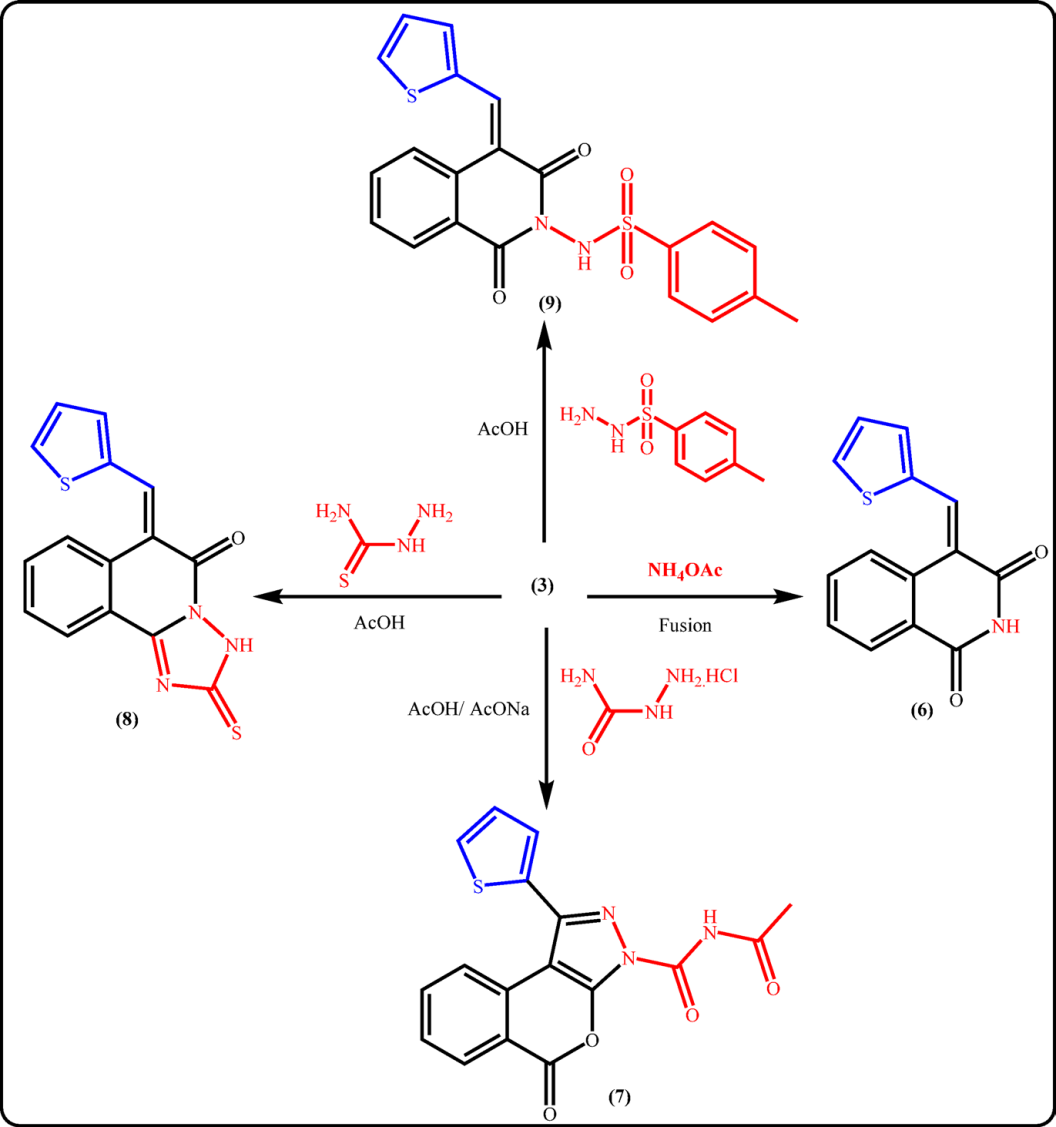
Reaction of 3 with ammonium acetate and some acid hydrazides.

In addition, this study was extended to explore the reaction of the unsaturated anhydride **3** with ammonium acetate and some acid hydrazides in an attempt to synthesis new isoquinolinone derivatives and related compounds. Thus, fusion of isochroman-1,3-dione **3** with ammonium acetate in an oil bath furnished the corresponding isoquinolinone derivative **6**. The characteristic absorption band for NH at 3172 cm^−1^ in IR spectrum of **6** alongside a broad signal at δ 11.52 ppm in ^1^H-NMR spectrum reinforce the suggested structure. Furthermore, refluxing **3** with semicarbazide hydrochloride in the presence of fused sodium acetate in glacial acetic acid yielded the unexpected heteroannelated compound which identified as isochromeno[3,4-c]pyrazole derivative **7 **(Fig. 8). Isolation of **7** can be proceeded *via* Aza-Michael addition reaction of *α*,*β*-unsaturated carbonyl, cyclization, followed by acylation. The IR spectrum of **7** exhibited υNH at 3189 cm^−1^ and carbonyl absorption bands at 1717 and 1672 cm^−1^ for a cyclic ester and amide, respectively. Moreover, ^1^H NMR spectrum was in consistent with the proposed structure.

 In contrast, when compound **3** was treated with thiosemicarbazide in boiling acetic acid, the heteroannelated 1,2,4-triazoloisoquinoline derivative **8** was obtained in 84% yield. Additionally, isoquinolinone derivative **9** was generated as green crystals in 66% yield through reaction of **3** with *p*-toluenesulfonohydrazide in refluxing acetic acid (Fig. 8). A strong evidence of structure 9 was gained from ^1^H- and ^13^C-NMR spectra which proved the result of the cyclization reaction.

### Density functional theory (DFT) investigation

#### Geometry optimization

Density Functional Theory (DFT) is an extremely efficacious theoretical technique with numerous applications. It can be used to establish compound’s thermodynamic and kinetic stability, obtain insights into mechanisms, achieve structural calculations, explore molecular interactions, and estimate the electronic and optical properties of molecules and atoms. Molecular geometry is the procedure that aims to regulate the low-energy molecular arrangement^55^ The synthetic compounds’ geometric properties were optimized using DFT at B3LYP/3-21G basis set. The final optimized molecular structures of molecules with the atom numbering planner are shown in Fig. 3.

#### Frontier molecular orbital (FMO)

**Fig. 9:**
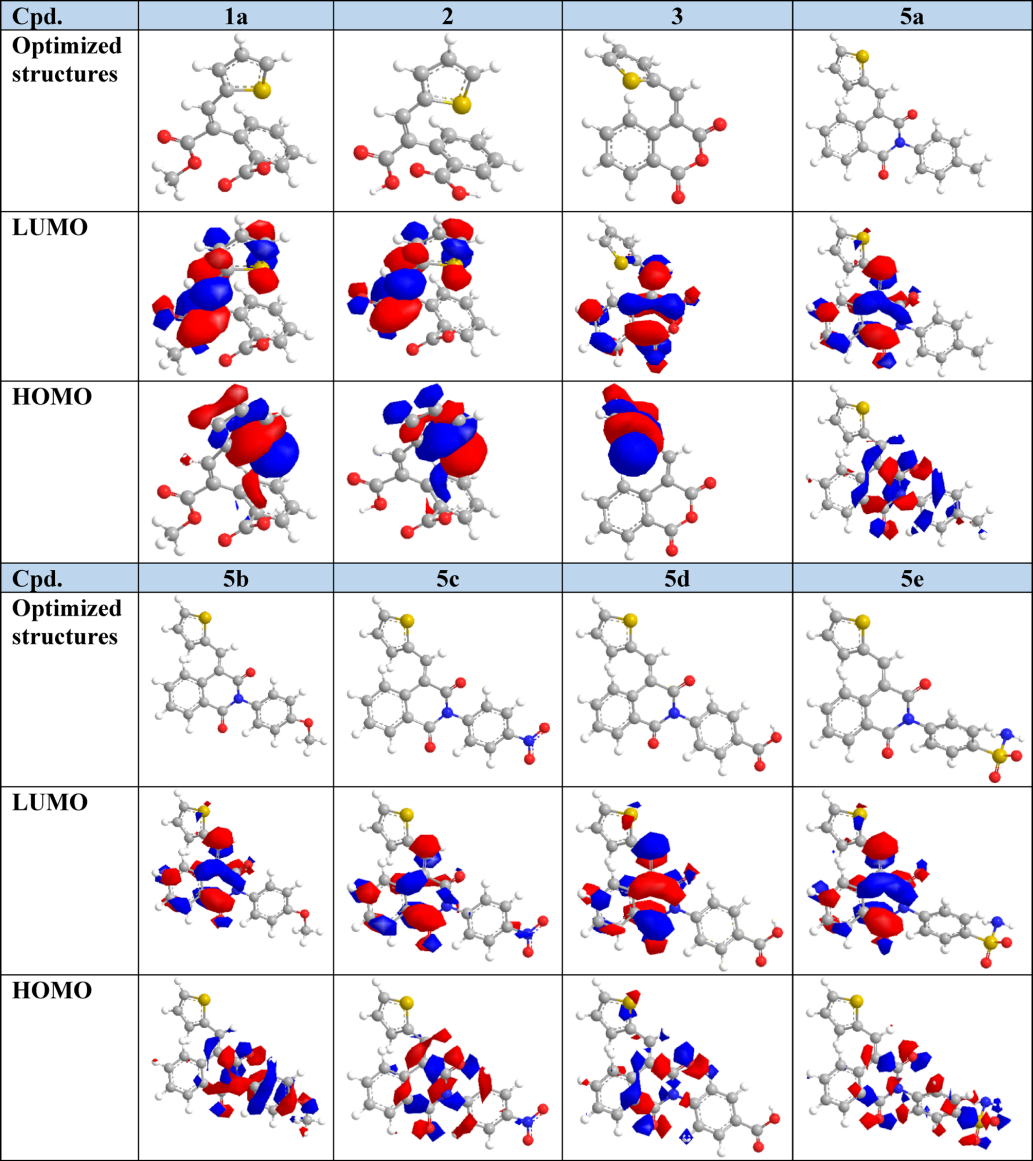

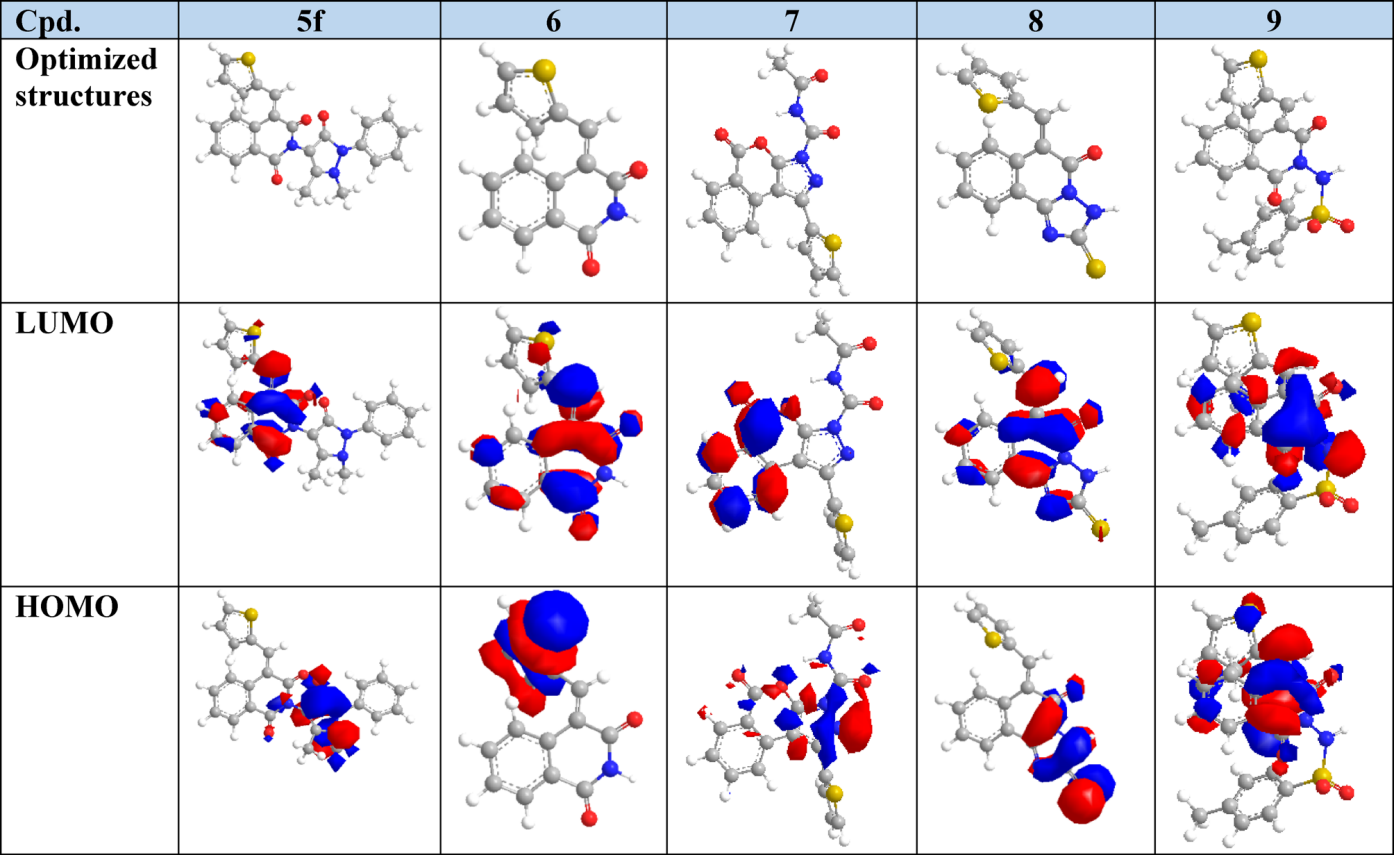
Optimized structures, HOMO, and LUMO for all prepared compounds. Atom color index: white H, grey C, blue N, yellow S, and red O.

Frontier Molecular Orbital (FMO) analysis plays a crucial role in analyzing the chemical molecule’s reactivity and stability by studying the energy levels. During molecular interactions, the main partners are the highest occupied molecular orbital (HOMO) and the lowest unoccupied molecular orbital (LUMO). These values used to establish the chemical reactivity and kinetic stability of molecules. The energy of LUMO energy level outline electron-accepting abilities while HOMO exhibits electron donating ability. In this study, the molecular geometry of all prepared thiophene-isoquinolinone derivatives was successfully optimized, producing local minima for these compounds. The HOMO and LUMO energy gap (ΔE) determines kinetic stability and chemical reactivity. A large ΔE value demonstrates high stability and low reactivity, whereas a smaller gap induces electronic charge transfer, making the molecule more reactive and polarized, also known as a “soft” molecule. In turn, a high ΔE represents a “hard” molecule with greater stability and lower reactivity^56^. The energies of FMOs (E_LUMO_, E_HOMO_) of the new molecules obtained are listed in (Table 1; Fig. 9).

In the case of HOMO, the charge density for compounds **1a**, **2**, **3** and **6** is accumulated at the thiophene moiety. In turn, for compounds **5a**-**5e**, the charge density is distributed in the isoquinolinone ring along with the aryl ring as well. While in compound **5f**, the HOMO is spread over the pyrazole unit.

The energy gaps (ΔE) between HOMO and LUMO are found in the range 1.81–6.91 eV. It was noted that compound **9** has a significantly lower ΔE of 1.81 eV whereas compound **7** has greater ΔE of 6.91 eV. The global chemical reactivity descriptors which include chemical potential (µ), hardness (η), softness (S), electrophilicity index (ω), electron affinity (A), ionization potential (*I*_P_), and electronegativity (χ) are responsible for identifying many of the fundamental chemical concepts^57^. These parameters are computed at the level of theory B3LYP/6–311 + + G (d, p)^58^. The values of these parameters for all synthesized compounds are displayed in Table 1.

**Table 1:** Global reactivity indices and energy level distribution of frontier orbitals.

Cpd.	*E	E_HOMO_	E_LUMO_	ΔE	µ	η	S	ω	A	I_*P*_	χ
**1a**	42.11	−10.772	−4.049	6.72	−7.40	3.36	0.29	8.14	4.04	10.77	7.40
**2**	22.82	−10.777	−4.021	6.75	−7.39	3.37	0.29	8.10	4.02	10.77	7.39
**3**	46.35	−11.439	−4.809	6.63	−8.11	3.31	0.30	9.93	4.80	11.43	8.11
**5a**	77.65	−10.685	−4.587	6.09	−7.63	3.04	0.32	9.57	4.58	10.68	7.63
**5b**	78.48	−10.462	−4.598	5.86	−7.52	2.93	0.34	9.65	4.59	10.46	7.52
**5c**	108.78	−10.761	−4.685	6.07	−7.72	3.03	0.33	9.83	4.68	10.76	7.72
**5d**	76.68	−11.212	−4.818	6.39	−8.01	3.19	0.31	10.05	4.81	11.21	8.01
**5e**	103.37	−11.088	−4.903	6.18	−7.99	3.09	0.32	10.33	4.90	11.08	7.99
**5f**	126.25	−8.906	−4.755	4.15	−6.82	2.07	0.48	11.23	4.75	8.90	6.82
**6**	3.29	−11.427	−5.054	6.37	−8.26	3.21	0.31	10.62	5.05	11.47	8.26
**7**	89.39	−10.629	−3.713	6.91	−7.16	3.45	0.28	7.42	3.71	10.62	7.16
**8**	48.97	−9.152	−4.874	4.27	−7.01	2.14	0.46	11.48	4.87	9.15	7.01
**9**	86.81	−5.802	−3.991	1.81	−4.89	0.90	1.11	13.28	3.99	5.80	4.89

According to these features, the chemical reactivity of the present compounds varies depending on their molecular structure. For instance, Hardness (η) and softness (S) are useful concepts for interpreting the reactivity of each compound. In comparison to other compounds, compound **9** with the lowest hardness of 0.9 eV and highest softness of 1.11 eV is the less stable and most active molecule; conversely, compound **7** with the highest hardness of 3.45 eV and lowest softness of 0.28 eV is the most stable and least active molecule. Further, electronegativity (χ) is used to illustrate the ability of a molecule to gain electrons^59^. As observed, compound **6** is the best electron acceptor due to its higher electronegativity value (χ = 8.26 eV) compared to all other compounds.

### Biological activity

#### Larvicidal assay

To evaluate insecticidal activity, thirteen compounds, twelve thiophene-isoquinolinone derivatives and the thiophene-based half-ester precursor molecule 1a, were tested against third-instar *Culex pipiens* larvae. The larvicidal bioassay showed strong activity across the series, with LC₅₀ values ranging from 0.004 to 28 µg/mL (Table 2). The precursor compound 1a had the strongest activity (LC₅₀ = 0.004 µg/mL) and served as the reference to calculate the toxicity index (TI) for the other compounds. The TI measures each compound’s potency compared to 1a, where higher TI values mean greater relative toxicity. Based on this measure, the thiophene-isoquinoline derivatives; 6 (LC₅₀ = 0.1 µg/mL; TI = 4), 5f (0.3 µg/mL; TI = 1.33), and 7 (1.85 µg/mL; TI = 0.21) were identified as the most potent compounds after 1a, making them strong candidates for controlling mosquito larvae.

All other derivatives, including compound 2 (14 µg/mL), 5a–5e (16.3–25.3 µg/mL), 8 (23.8 µg/mL), and 9 (16.4 µg/mL), showed moderate larvicidal activity. Even the least effective compound, 5b (28 µg/mL), was over ten times more potent than chlorpyrifos (LC₅₀ = 293.8 µg/mL; TI = 0.001), a common organophosphate insecticide. These results show that the thiophene-isoquinolinone structure keeps significant activity across a wide range of structural changes.

The dose-response data for each compound were analyzed using probit regression. This is a well-known method for modeling biological responses to different doses^60–64^. Prior to analysis, mortality data were corrected using Abbott’s formula^65^, based on the average mortality observed in the negative control group, which consisted of larvae exposed only to the solvent carrier (DMF) diluted in water. This correction ensures that the calculated effects reflect compound-induced mortality rather than background or solvent-related effects. Probit analysis estimates the median lethal concentration (LC₅₀) and provides 95% confidence intervals by fitting a cumulative normal distribution function to the mortality data observed. This method allows for accurate measurement of compound potency and helps compare toxicity across similar compounds.

To check how well the probit model fits each compound, we used the Pearson chi-square (χ²) test. This test looks at the difference between the observed mortality rates and those expected from the model. A χ² value lower than the critical value of 7.8, along with a p-value greater than 0.05, suggests that the differences between observed and predicted responses are not significant. This confirms that the model is appropriate.

In this study, all compounds showed χ² values below the critical threshold and had p-values above 0.05. These findings confirm the reliability of the LC₅₀ estimates and show that the probit regression effectively described the observed dose-response relationships. Therefore, the LC₅₀ values and the toxicity indices (TI) calculated in relation to the most potent compound (1a) are strong and suitable for comparing toxicological effects.

In all bioassays, treated larvae consistently showed neurotoxic symptoms such as hyperactivity, tremors, convulsions, and paralysis. These symptoms closely resembled those caused by the acetylcholinesterase (AChE) inhibitor, chlorpyrifos. This symptom pattern, along with the structural similarities of the tested compounds to organophosphates and neonicotinoids, suggests a possible mechanism involving disruption of cholinergic signaling, likely through AChE inhibition or interaction with nicotinic acetylcholine receptors (nAChRs). Previous studies showing thiophene-based compounds as AChE inhibitors further support the role of the thiophene group in causing the observed neurotoxicity^29^.

**Table 2:** Larvicidal activity of the precursor compound 1a and twelve thiophene-isoquinolinone derivatives against third-instar *Culex pipiens* larvae, compared to chlorpyrifos, at 24 h post-treatment. Data are expressed as LC₅₀ values with 95% confidence intervals.

Compound No.	LC_50_ (µg/mL)	*r* ^2^	χ^2^cal./Tab._(7.8)_	χ^2^ *P*-value	Toxicity index
1a	0.004	0.78	0.9	0.88	100
2	14	0.97	3.7	0.49	0.028
3	27	0.95	7.7	0.08	0.0141
5a	25.3	0.96	6.5	0.21	0.015
5b	28	0.98	7.1	0.19	0.0142
5c	24.6	0.95	6	0.23	0.016
5d	24.1	0.96	7.2	0.11	0.0165
5e	16.3	0.97	7.3	0.07	0.024
5f	0.3	0.97	0.2	0.86	1.33
6	0.1	0.95	0.4	0.91	4
7	1.85	0.95	5.1	0.29	0.21
8	23.8	0.95	7.4	0.18	0.0168
9	16.4	0.97	4.7	0.66	0.0243
Chlorpyrifos	293.8	0.98	2.2	0.92	0.001

#### Molecular docking assessment

Molecular docking simulations were conducted to evaluate the interactions of 12 newly synthesized thiophene–isoquinolinone derivatives, along with the precursor compound 1a, with two key mosquito neural targets: acetylcholinesterase (AChE) and the nicotinic acetylcholine receptor (nAChR). These targets were selected based on neurotoxic symptoms observed in larvicidal bioassays, which suggested disruption of neural signaling. Their prioritization was further supported by the structural similarity of the compounds to organophosphates and neonicotinoids, which exert toxicity through AChE inhibition and nAChR activation, respectively.

AChE is critical for terminating synaptic transmission by hydrolyzing acetylcholine (ACh). Inhibition of AChE, such as by chlorpyrifos, leads to ACh accumulation, resulting in sustained neuronal stimulation, paralysis, and death^66,67^. In contrast, neonicotinoids (e.g., thiamethoxam, imidacloprid) act as ACh mimics that activate nAChRs, causing persistent ion channel opening and neural hyperexcitation, ultimately disrupting nervous system function^68–70^.

Due to the absence of resolved *Culex pipiens* AChE and nAChR structures in the Protein Data Bank (PDB), surrogate models were used. For AChE, we selected the crystal structure of an insecticide-resistant mutant from *Anopheles gambiae* (PDB ID: 6ARY), representing a biologically relevant variant for assessing resistance-overcoming potential. For nAChR, a homology model was built using the *Aedes aegypti* Noppera-bo receptor as a template, yielding a high-confidence structure with a GMQE score of 0.91 (Supplementary Data).

Molecular docking was performed to assess the binding affinities and interaction profiles of the synthesized compounds against AChE. The redocking of the co-crystallized ligand BT7 validated the docking protocol, yielding an RMSD of 0.78 Å and a score of − 5.34 kcal/mol. Chlorpyrifos was also docked as a reference. Most derivatives showed stronger binding affinities, ranging from − 6.10 to − 8.47 kcal/mol (Table 3). The top-scoring compounds were compound 9 (–8.47 kcal/mol), 5c (–7.60 kcal/mol), and 5 d (–7.44 kcal/mol). Precursor 1a displayed a binding score of − 6.72 kcal/mol, slightly better than chlorpyrifos (–6.62 kcal/mol). Although compounds 3, 6, and 8 exhibited lower scores than chlorpyrifos, they still outperformed BT7 in predicted affinity.

In this redocked pose, BT7 maintained an interaction with SER280, consistent with the crystallographic pose, but formed a new contact with TYR282, which was not present in the original structure. Notably, several key interactions seen in the crystal structures such as those with ALA361, SER360, GLY279, GLU359, and TRP245—were absent in the redocked configuration. This discrepancy highlights the limitations of rigid docking, particularly its inability to account for protein flexibility and solvation effects.

Despite the limitations of rigid docking, most of the synthesized compounds displayed partial overlap in binding mode with the original co-crystallized BT7 ligand, particularly through interactions with residues such as SER360, SER280, GLY279, GLU359, and TRP245, all located within the catalytic gorge of AChE and commonly associated with ligand recognition and inhibition. Notably, GLU359, a key residue in the BT7 crystallographic pose, was also engaged by chlorpyrifos as well as derivatives 5b, 5c, 5f, 6, and 8, further supporting a consistent binding pattern across both experimental and reference ligands (Table 3 & S. Data File 1).

Additionally, a subset of compounds—including 1a, 2, 5a, 5b, 5e, 5f, and 7—formed interactions with SER280, a residue involved in both the crystallographic pose and retained in the redocked BT7 configuration. This variability in interaction patterns highlights flexibility in how these compounds accommodate within the active site, with SER280 and GLU359 emerging as a potentially adaptive anchoring point under docking conditions. The frequent interactions observed between the synthesized derivatives and key residues such as GLU359 and SER280—components of the known SER–HIS–GLU catalytic triad in human AChE—further support the hypothesis that these compounds may act as effective AChE inhibitors through engagement with catalytically essential sites^29,61,64^.

**Fig. 10:**
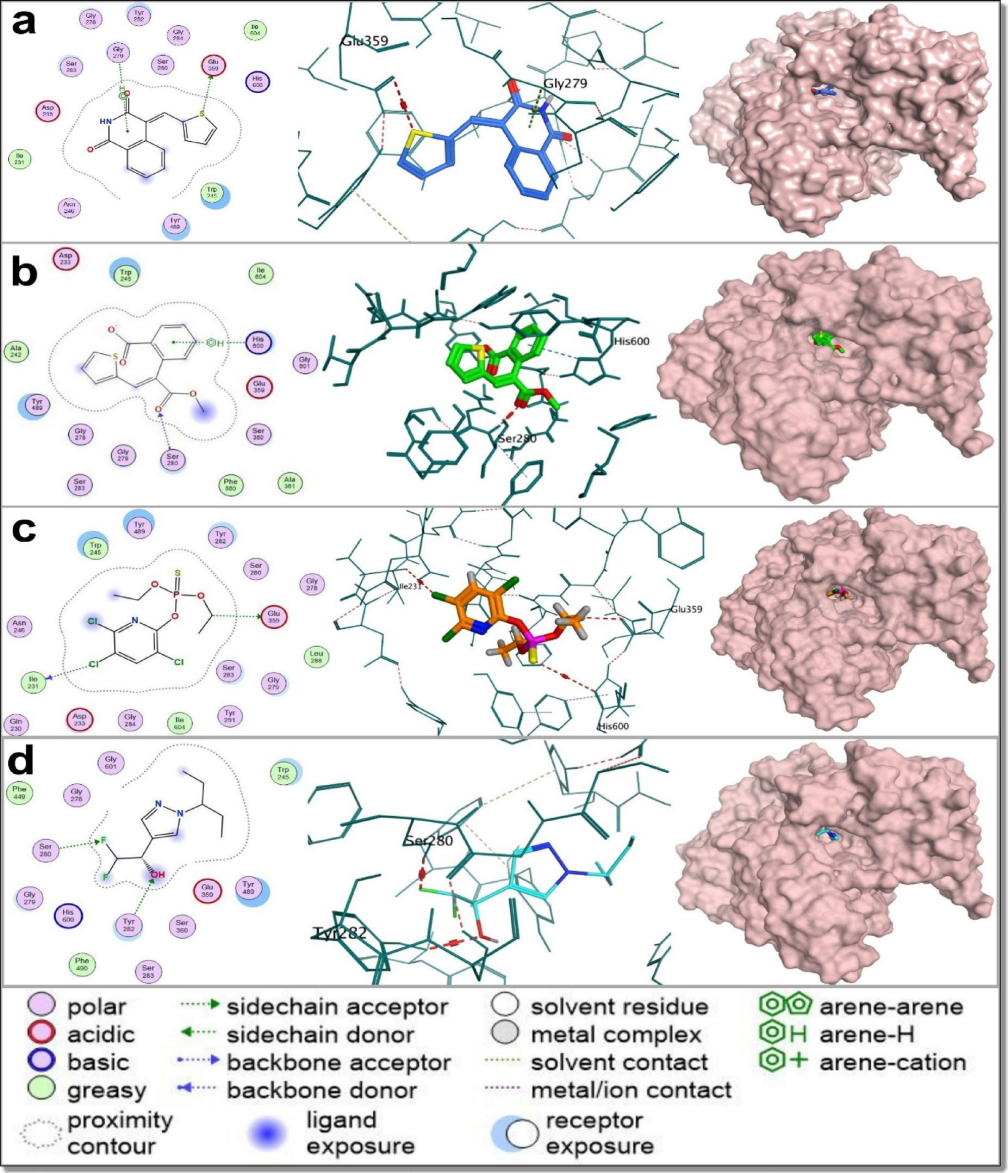
Comparative 2D and 3D interaction diagrams illustrating the binding profiles of key ligands within the active site of the insecticide-resistant acetylcholinesterase (AChE) variant from *Anopheles gambiae* (PDB ID: 6ARY). Panel (**a**) shows compound 6, the most potent thiophene–isoquinolinone derivative in the larvicidal assay; panel (**b**) shows the precursor compound 1a; panel (**c**) shows chlorpyrifos; and panel (**d**) shows the redocked co-crystallized ligand BT7. Compound 6 interacts with GLU359—a residue also engaged by chlorpyrifos—while compound 1a interacts with SER280, shared with BT7. Both residues are part of the BT7 co-crystallized pose and the conserved catalytic triad of human AChE, highlighting key overlaps with known AChE inhibitors and supporting the potential of compounds 6 and 1a to inhibit AChE via a conserved and functionally relevant binding mode.

Notably, compound 6 and the precursor 1a were among the most potent compounds in the larvicidal bioassay, exhibiting the lowest LC₅₀ values in the series. At the molecular level, compound 6 shared a key interaction with chlorpyrifos at GLU359, while 1a overlapped with the redocked BT7 by forming a hydrogen bond with SER280. Both GLU359 and SER280 are present in the original crystallographic binding pose of BT7 and are components of the conserved catalytic triad (SER–HIS–GLU) in acetylcholinesterase, highlighting the functional significance of these interactions in supporting the compounds’ inhibitory potential (Fig. 10).

The observed overlap in binding site engagement among the derivatives, BT7 (both native and redocked), and chlorpyrifos—particularly at residues critical for catalytic activity—strongly suggests that these compounds share a common inhibitory mechanism. Taken together, these results support the high potential of thiophene-isoquinolinone derivatives to function as effective AChE inhibitors through conserved and biologically relevant interactions.

**Fig. 11:**
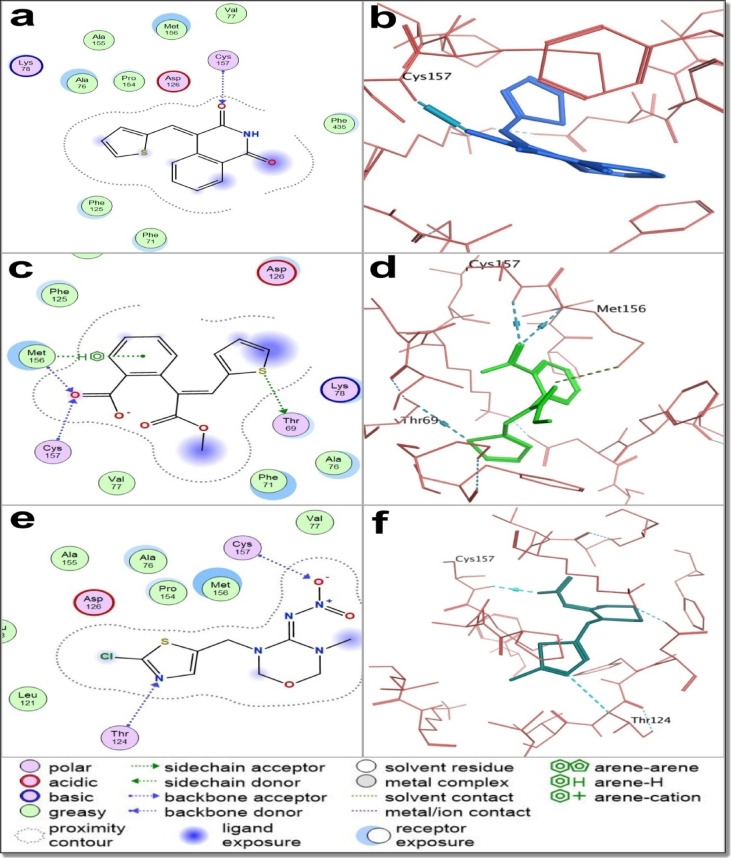
2D and 3D interaction diagrams of selected compounds docked into the homology-modeled *Culex pipiens* nicotinic acetylcholine receptor (nAChR). Panels (**a**) and (**b**) show compound 6, the most potent thiophene-isoquinolinone hybrid based on larvicidal bioassay results. Panels (**c**) and (**d**) depict the precursor compound 1a, which exhibited the highest overall larvicidal potency among all tested compounds. Panels (**e**) and (**f**) illustrate the reference neonicotinoid agonist thiamethoxam. Both compounds 6 and 1a form hydrogen bonds with CYS157, a key interaction also observed in thiamethoxam. The shared engagement with CYS157 suggests that compounds 6 and 1a may act as functional mimics of neonicotinoid insecticides, supporting their proposed mode of action through nAChR modulation.

To explore dual-target activity, docking was also performed against the nAChR homology model. Predicted binding affinities ranged from − 5.38 to − 7.30 kcal/mol (Table 4), with compound 9 (–7.30 kcal/mol), 5e (–6.90 kcal/mol), and 5f (–6.81 kcal/mol) ranking highest—comparable to thiamethoxam (–7.11 kcal/mol). Interaction analysis revealed consistent engagement of residues such as CYS157, MET156, TYR159, and ASP126, with CYS157 emerging as a conserved anchoring point across nearly all active compounds and the reference ligand. Compounds 1a and 6 both formed hydrogen bonds with CYS157, supporting a neonicotinoid-like binding mode (Fig. 11 & Supplementary Data).

RMSD values for all docked ligands ranged from 0.62 to 1.93 Å for AChE and 0.78 to 1.95 Å for nAChR, with BT7 redocking confirming protocol reliability. These values fall within acceptable thresholds, indicating stable and plausible docking poses for subsequent interaction analysis.

Taken together, docking results show that the thiophene–isoquinolinone derivatives effectively target both AChE and nAChR through conserved interactions at catalytically and functionally critical residues. The overlap of binding profiles with known insecticides—particularly interactions involving GLU359 and SER280 in AChE, and CYS157 in nAChR—supports a potential dual mechanism of neurotoxic action. RMSD analyses affirmed stable binding poses across both targets, reinforcing the reliability of the docking predictions. While these findings offer insights that match the observed larvicidal and neurotoxic effects, they remain predictions and should be confirmed through biochemical assays, such as AChE inhibition studies and receptor-based functional tests, in future research.

The strong correlation between molecular docking outcomes and larvicidal bioassay results reinforces the role of acetylcholinesterase (AChE) and the nicotinic acetylcholine receptor (nAChR) as principal neurotoxic targets of the synthesized thiophene-isoquinolinone derivatives. While docking scores provide valuable insights into receptor-ligand affinity, it is important to recognize that binding energy alone does not fully explain in vivo insecticidal potency. Variations in LC₅₀ values among compounds with comparable docking profiles may arise from factors such as metabolic stability, absorption, bioavailability, and distribution within the insect system^60,64^. Nonetheless, the convergence of rational structural design, high in silico receptor affinity, and potent biological activity strongly supports AChE and nAChR as central mediators of the observed neurotoxicity.

In conclusion, the synthesized thiophene-isoquinolinone hybrids exhibit key structural and functional elements that align closely with established neuroactive insecticides. Their ability to effectively engage both AChE and nAChR through complementary interaction modes underscores their potential as dual-target insecticidal agents. This dual mechanism not only enhances efficacy but also offers a promising strategy to overcome existing resistance mechanisms, positioning these compounds as strong candidates for the development of next-generation mosquito control agents.

**Table 3:** Molecular Docking scores and interaction profiles of 12 synthesized thiophene–isoquinoline derivatives and their precursor compound, 1a (a thiophene-based half ester), were evaluated against acetylcholinesterase (AChE; PDB ID: 6ARY). Results were compared with the reference insecticide Chlorpyrifos and the redocked co-crystallized ligand BT7.

Code	S	RMSD	Residues	Interaction	Distance	E (kcal/mol)
1a	−6.72	1.38	SER 280 (B)	H-acceptor	3.12	−1.9
			HIS 600 (B)	pi-H	4.01	−0.5
2	−6.56	0.82	GLY 279 (B)	H-acceptor	2.91	−1.4
			SER 280 (B)	H-acceptor	3.04	−4.5
			HIS 600 (B)	Ionic	2.93	−5
			HIS 600 (B)	pi-H	4.06	−0.5
3	−6.10	1.93	SER 360 (B)	H-donor	3.94	−1
5a	−7.33	1.05	SER 280 (B)	H-acceptor	2.87	−0.7
5b	−7.19	1.24	GLU 359 (B)	H-donor	3.2	−0.6
			SER 280 (B)	H-acceptor	2.91	−1
			TRP 245 (B)	H-pi	3.69	−0.5
5c	−7.60	1.18	GLU 359 (B)	H-donor	4.04	−0.6
			VAL 232 (B)	H-acceptor	3.34	−0.6
			VAL 232 (B)	H-acceptor	3.12	−0.5
			GLY 279 (B)	pi-H	4.34	−0.5
			TYR 489 (B)	pi-H	4.7	−0.5
5d	−7.44	1.27	GLU 359 (B)	H-donor	4.05	−0.6
			VAL 232 (B)	H-acceptor	3.32	−1.2
			ASN 246 (B)	H-acceptor	3.57	−0.6
			VAL 232 (B)	H-acceptor	3.1	−0.9
5e	−7.12	1.54	PHE 490 (B)	H-donor	2.94	−0.8
			SER 280 (B)	pi-H	3.9	−0.7
			PHE 490 (B)	pi-H	4.2	−0.6
5f	−7.43	1.83	GLU 359 (B)	H-donor	3.47	−0.6
			SER 280 (B)	H-acceptor	3.19	−0.7
6	−6.12	1.31	GLU 359 (B)	H-donor	3.3	−0.6
			GLY 279 (B)	pi-H	4.12	−0.6
7	−7.37	1.03	GLY 279 (B)	H-acceptor	3.1	−1.3
			SER 280 (B)	H-acceptor	2.88	−1.8
8	−6.31	0.62	GLU 359 (B)	H-donor	2.9	−0.9
			GLY 279 (B)	pi-H	3.69	−0.6
			GLY 279 (B)	pi-H	4.48	−0.5
9	−8.47	1.47	GLY 279 (B)	pi-H	4.35	−1
			GLY 601 (B)	pi-H	4.71	−0.6
Chlorpyrifos	−6.62	1.79	ILE 231 (B)	H-donor	3.50	−0.6
			GLU 359 (B)	H-donor	3.31	−0.5
Redocked Co-Crystallized ligand	−5.34	0.78	SER 280 (B)	H-acceptor	2.77	−0.6
			TYR 282 (B)	H-acceptor	2.96	−1.3

**Table 4:** Molecular Docking scores and interaction profiles of 12 synthesized thiophene–isoquinoline derivatives and the precursor compound 1a (a thiophene-based half ester) were evaluated against the nicotinic acetylcholine receptor (nAChR) of culex pipiens and compared to the reference insecticide thiamethoxam.

Code	S	RMSD	Residues	Interaction	Distance	E (kcal/mol)
1a	−5.97	1.01	THR 69 (A)	H-donor	3.44	−1
			MET 156 (A)	H-acceptor	3.29	−0.7
			CYS 157 (A)	H-acceptor	3.5	−0.9
			MET 156 (A)	pi-H	3.7	0
2	−6.16	1.22	MET 156 (A)	H-acceptor	3.27	−0.7
			CYS 157 (A)	H-acceptor	3.44	−1.6
			LYS 78 (A)	H-acceptor	3.39	−0.8
			PRO 154 (A)	H-acceptor	3.31	−0.5
			LYS 78 (A)	pi-H	4.08	−0.5
3	−5.45	1.13	THR 69 (A)	H-donor	3.01	−0.9
			CYS 157 (A)	H-acceptor	3.47	−0.8
5a	−5.38	1.24	LYS 434 (A)	H-donor	3.97	−0.6
5b	−6.16	1.07	VAL 161 (A)	H-acceptor	3.34	−1.2
5c	−6.53	0.78	CYS 157 (A)	H-acceptor	2.94	−2.9
			TYR 159 (A)	H-acceptor	3.34	−1.5
5d	−6.33	1.95	CYS 157 (A)	H-donor	3.33	−1.8
			CYS 157 (A)	H-acceptor	3.3	−1.6
			ASP 126 (A)	H-acceptor	3.17	−2.3
5e	−6.90	1.53	CYS 157 (A)	H-acceptor	2.92	−3.4
			TYR 159 (A)	H-acceptor	3.25	−1.7
5f	−6.81	1.33	CYS 157 (A)	H-donor	3.51	−1.5
6	−5.50	1.85	CYS 157 (A)	H-acceptor	2.91	−4
7	−5.70	1.60	VAL 77 (A)	H-acceptor	3.45	−0.6
			CYS 157 (A)	H-acceptor	2.89	−4
			PHE 435 (A)	pi-H	3.61	−0.7
8	−5.93	1.83	CYS 157 (A)	H-acceptor	3.2	−0.8
9	−7.30	1.63	MET 156 (A)	H-acceptor	3.36	−0.5
			LYS 78 (A)	Ionic	3.43	−2.2
Thiamethoxam	−7.11	1.38	CYS 157 (A)	H-acceptor	3.35	−0.8
			THR 124 (A)	H-acceptor	3.61	−0.5

#### Molecular dynamics simulations

Molecular dynamics (MD) simulations were employed to validate and extend the molecular docking results by providing a more realistic and time-resolved assessment of ligand–target interactions under near-physiological conditions. While docking offers valuable initial insights into potential binding poses and affinities, it represents a static model that does not account for receptor flexibility, solvent dynamics, or temporal fluctuations in molecular interactions. Therefore, MD simulations serve as a crucial complementary approach, enabling the evaluation of complex stability, interaction persistence, and the conformational behavior of both ligand and protein over time^60,62,71^.

The starting molecule, 1a—which achieved the lowest LC₅₀—and the most potent thiophene–isoquinoline compound, 6, were selected for molecular dynamics (MD) simulations based on their superior docking performance with acetylcholinesterase (AChE) compared to the nicotinic acetylcholine receptor (nAChR). The aim was to assess whether their docked poses within the AChE active site remained stable over a 100 ns simulation and to compare their binding behavior with that of chlorpyrifos and the redocked co-crystallized AChE inhibitor, BT7.

**Fig. 12:**
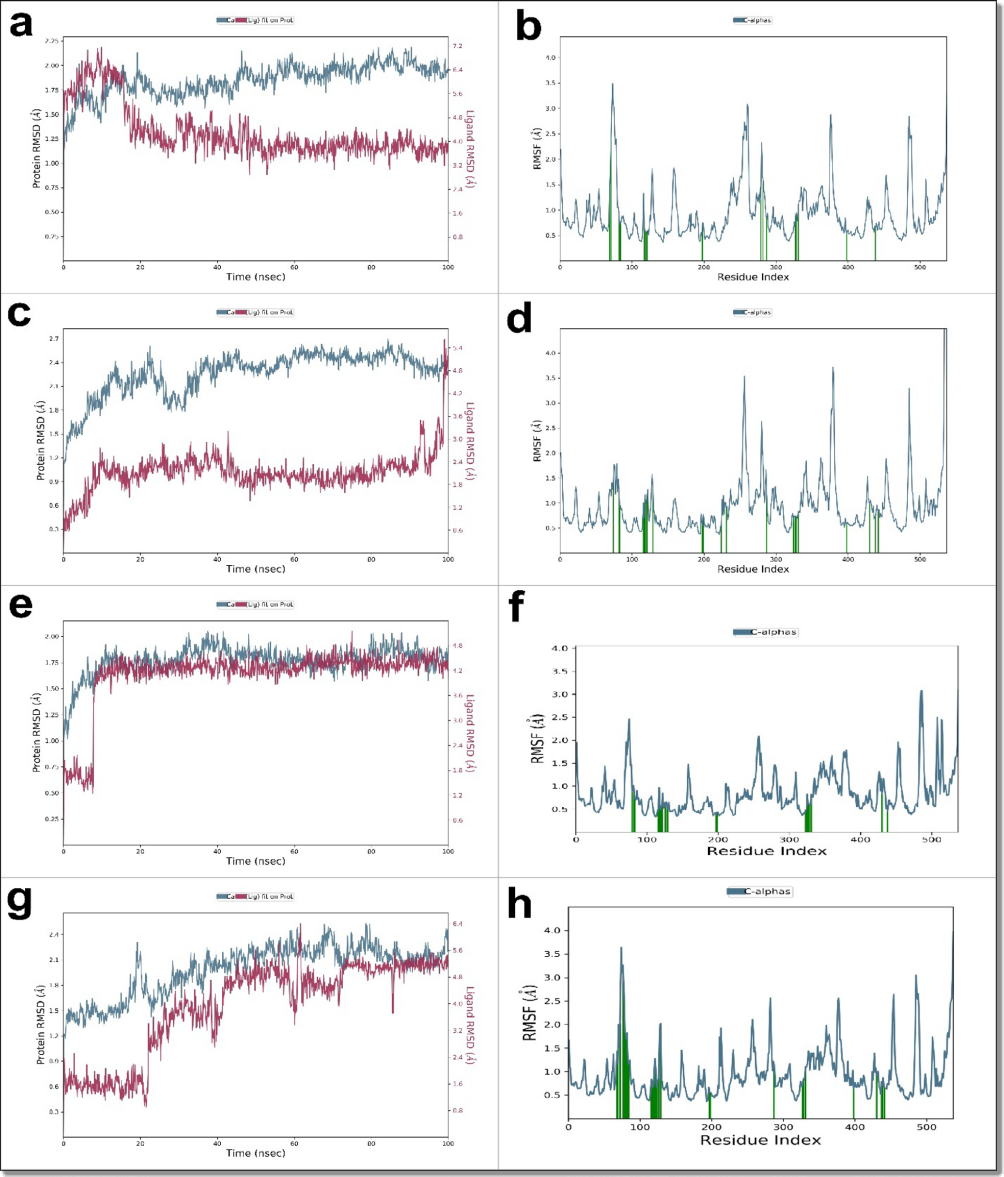
Root mean square deviation (RMSD) and root mean square fluctuation (RMSF) analysis of AChE (PDB ID: 6ARY) in complex with different ligands over a 100 ns MD simulation. Panels (**a–h**) show backbone and ligand RMSD (left) and RMSF (right) for AChE bound to compound 6, compound 1a, chlorpyrifos, and the redocked co-crystallized ligand BT7, respectively. All complexes exhibited stable binding with minimal fluctuations, confirming their overall structural stability. Minor ligand-dependent variations in residue flexibility were observed, supporting the stable and reliable binding of compounds 1a and 6 within the AChE active site.

The MD analysis included Root Mean Square Deviation (RMSD) to evaluate overall complex stability, Root Mean Square Fluctuation (RMSF) to examine residue flexibility, and ligand–receptor interaction profiling to determine the persistence of hydrogen bonds, hydrophobic contacts, and ionic interactions **(Supplementary Data)**. While RMSD and RMSF values remained within stable ranges for all ligands, suggesting no major conformational changes in the AChE–ligand complexes **(**Fig. 12**)**, these parameters alone did not fully account for differences in binding quality among the compounds.

**Fig. 13:**
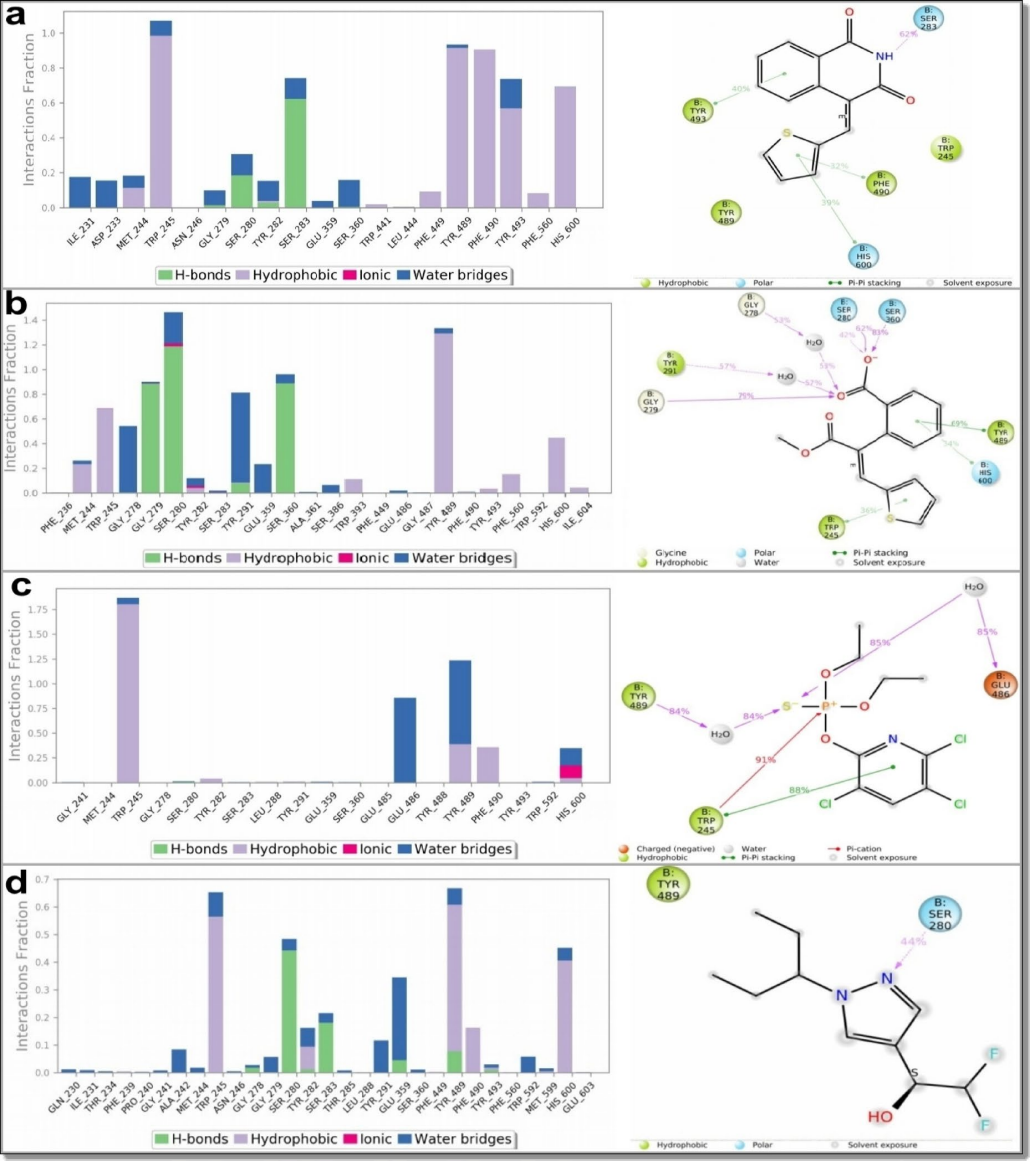
Molecular dynamics (MD) simulation analyses showing protein–ligand interaction histograms and contact frequencies for (**a**) compound 6, (**b**) the precursor molecule 1a, (**c**) chlorpyrifos, and (**d**) the redocked co-crystallized ligand BT7, all in complex with the crystal structure of an insecticide-resistant acetylcholinesterase (AChE) mutant from *Anopheles gambiae*. The histograms illustrate the stability and frequency of key interactions, including hydrogen bonds, hydrophobic, and ionic contacts. Compounds 6 and 1a maintained strong and consistent interactions throughout the simulation, overlapping significantly with those of BT7. In contrast, chlorpyrifos formed fewer and less stable contacts, highlighting the enhanced binding stability—and potential insecticidal efficacy—of compounds 6 and 1a against resistant AChE.

Ligand interaction histograms **(**Fig. 13**)** revealed distinct differences in binding persistence and interaction networks. Compounds 1a and 6, as well as the redocked ligand BT7, engaged several conserved active site residues—TRP245, SER280, TYR489, and HIS600—through stable hydrogen bonding and hydrophobic interactions. Notably, compound 1a formed the highest number of persistent interactions, surpassing both compound 6 and BT7. This stable and extensive interaction network correlates with its superior inhibitory activity. Compound 6 also maintained consistent contacts with key residues, supporting its effectiveness as an AChE inhibitor. In contrast, chlorpyrifos displayed fewer and less stable interactions, with limited engagement of critical residues, aligning with its lower potency.

These findings highlight the enhanced binding capability and inhibitory strength of compounds 1a and 6 and underscore the improved predictive power of MD simulations over molecular docking for evaluating ligand–target interactions. The dynamic interaction patterns observed for 1a and 6 suggest more stable and functionally favorable binding modes, consistent with their superior bioassay performance. Unlike docking, which provides only static binding scores, MD simulations offer a mechanistic explanation for their higher larvicidal potency relative to chlorpyrifos.

In summary, MD simulations validated the docking results and confirmed that the lead compound 1a and the potent thiophene–isoquinoline hybrid 6 form more stable and effective complexes with AChE than chlorpyrifos. Both compounds successfully reproduced key interactions observed in the co-crystallized ligand BT7 of AChE (PDB ID: 6ARY), supported by lower ligand RMSD values, reduced RMSF at critical active site residues, and persistent intermolecular contacts throughout the simulation. Notably, 1a—bearing a thiophene-based half ester scaffold—exhibited the strongest larvicidal activity and serves as a representative lead for a broader class of structurally diverse thiophene–isoquinoline derivatives. The structural flexibility of the precursor compound 1a enables the synthesis of a wide range of thiophene–isoquinoline derivatives with diverse functional features, offering a strategic advantage in mitigating resistance. Rather than depending on a single compound, the diversity within this chemical class enhances the likelihood of overcoming resistance mechanisms in *Culex pipiens* populations. Altogether, these findings highlight the thiophene–isoquinoline scaffold as a promising foundation for developing next-generation larvicidal agents targeting insecticide-resistant mosquito vectors.

#### Structure activity relationship (SAR)

The structural design of the synthesized thiophene-isoquinolinone derivatives aimed to improve insecticidal activity by targeting two neural receptors: acetylcholinesterase (AChE) and nicotinic acetylcholine receptors (nAChRs). All compounds have a thiophene core and an isoquinolinone scaffold, which are two heterocycles known for their bioactivity. This creates a rigid, conjugated framework that allows π-π stacking and hydrophobic interactions within receptor sites. Previous studies demonstrated that thiophene-based structures could inhibit AChE. This supports their role as a primary pharmacophore.

The structure–activity relationship (SAR) analysis of the thiophene-derived compounds (1a–9) reveals a consistent trend in how specific structural modifications influence their cytotoxic activity, as indicated by LC₅₀ values. The parent compound, 1a, contains an α,β-unsaturated ester (a Michael acceptor), a carboxylic acid group, and a thiophene ring at the β-position. It exhibited remarkably potent cytotoxic activity (LC₅₀ = 0.004 µg/mL). This pronounced activity is attributed to the presence of the highly electrophilic Michael acceptor, which can react with nucleophilic biological targets, potentially through covalent bonding. Additionally, the molecule’s planar, conjugated system likely facilitates effective cellular penetration and favorable interactions with biological macromolecules. The exceptional potency of 1a, as identified in the SAR analysis, is further supported by molecular docking results, which demonstrate direct binding interactions with SER280 in acetylcholinesterase (AChE) and CYS157 in nicotinic acetylcholine receptor (nAChR). These interactions with key nucleophilic residues support the compound’s capacity to form stable, possibly covalent, bonds at critical binding sites, reinforcing its potential mechanism of action and explaining its superior cytotoxicity.

When the methyl ester group in 1a is hydrolyzed to form the dicarboxylic acid derivative 2, the LC₅₀ drops significantly to 14 µg/mL, indicating a major loss in potency. This drop can be explained by the increased hydrophilicity and possible reduction in cell permeability, along with the decreased electrophilic character of the molecule. Further transformation to the cyclic anhydride structure in compound 3 results in an LC₅₀ of 27 µg/mL, showing a continued decrease in activity. The rigid, fused ring structure of 3 reduces flexibility and likely limits the compound’s ability to act like the parent molecule.

Reaction of compound 3 with various arylamines produced a series of imide-containing isoquinoline-1,3-dione derivatives (5a–5f) that showed moderate but varied cytotoxic activity. Compounds 5a and 5b, which had electron-donating groups (p-tolyl and 4-methoxyphenyl), displayed relatively low activity (LC₅₀ = 25.3 and 28 µg/mL, respectively). This suggests that higher electron density around the imide group decreases electrophilicity and reactivity with biological targets. On the other hand, derivatives with electron-withdrawing or polar substituents, such as 5c (4-nitrophenyl, LC₅₀ = 24.6 µg/mL), 5 d (carboxyphenyl, LC₅₀ = 24.1 µg/mL), and especially 5e (sulfonamide, LC₅₀ = 16.3 µg/mL), showed modest improvements in activity. The increased activity of 5e may be due to the additional hydrogen bonding interactions and higher polarity from the sulfonamide group, which could enhance biological binding.

A notable improvement in activity was found in compound 5f, which includes a pyrazolone group. This compound had an LC₅₀ of 0.3 µg/mL, nearly equal to the potency of the parent compound 1a. The pyrazolone ring restores a conjugated electron-deficient system, possibly regaining a Michael-acceptor-like character while benefiting from more aromatic stabilization and potential hydrogen-bonding interactions. This indicates that restoring conjugation and electronic balance around the isoquinoline-1,3-dione core is crucial for strong cytotoxicity.

Further transformation of compound 3 yielded a series of heterocyclic analogs (6–9) through condensation with ammonia, semicarbazide, thiosemicarbazide, and sulfonohydrazide, respectively. Among these, compound 6, a structurally minimal isoquinolinone derivative synthesized via fusion with ammonium acetate, exhibited notably strong cytotoxic activity (LC₅₀ = 0.1 µg/mL). This suggests that a compact fused ring system, which preserves molecular planarity and incorporates a hydrogen bond-donating NH group, can significantly enhance biological activity. This observation is strongly supported by molecular dynamics simulations, which revealed that the NH group in compound 6 forms a highly persistent hydrogen bond with Ser283 in the AChE active site, maintained for 62% of the simulation time. This stable interaction likely contributes to the compound’s potent binding affinity and biological effect. Similarly, compound 7, which features a fused pyrazolone ring formed through condensation of compound 3 with semicarbazide, retained substantial cytotoxic activity (LC₅₀ = 1.85 µg/mL), highlighting the beneficial effect of increased molecular rigidity and extended aromatic character. Importantly, the carbonyl group introduced via the pyrazolone ring, derived from the acetyl group of semicarbazide, plays a key role in binding. Docking studies reveal that this newly introduced carbonyl forms stable hydrogen bonds with SER280 and GLY279 in the AChE active site, while additional interaction with CYS157 in nAChR suggests a dual contribution to binding affinity. These observations support the conclusion that structural elaboration at this position not only enhances conformational stability but also provides new functional groups capable of engaging essential residues, thereby sustaining potent biological activity.

On the other hand, compound 8, which includes a fused triazolo ring via thiosemicarbazide, was significantly less active (LC₅₀ = 23.8 µg/mL), even though it is structurally similar to other active heterocycles. This might be due to increased steric hindrance or the electronic nature of the triazole, which could disrupt molecular recognition or lower membrane permeability. Compound 9, a sulfonohydrazide derivative, showed moderate activity (LC₅₀ = 16.4 µg/mL). It likely benefits from the sulfonamide group’s polarity and hydrogen-bonding abilities, just like compound 5e.

In summary, SAR trends suggest that the highest activity relates to structures that keep or mimic the electrophilic, conjugated α,β-unsaturated system found in 1a. Changes that add rigidity or remove this electrophilicity, such as cyclization to anhydride 3 or modifying with less reactive arylamines (5a–5d), usually lower activity. However, carefully adding heterocycles, especially those that restore conjugation and polarity (5f, 6, 7), can recover or even boost cytotoxic potency. Polar functional groups like sulfonamides increase activity somewhat by allowing more hydrogen bonding. In contrast, bulky or strongly electron-donating groups usually make it less effective. This analysis offers a clear guide for creating future analogs that balance electrophilicity, conjugation, and polarity to achieve high bioactivity.

## Materials and methods

### Chemistry

All melting points were measured on a Griffin and George melting-point apparatus (Griffin & Georgy Ltd., Wembley, Middlesex, UK) and are uncorrected. IR spectra were recorded on Pye Unicam SP1200 spectrophotometer (Pye Unicam Ltd., Cambridge, UK) by using the KBr wafer technique. ^1^H-NMR spectra were determined on a Varian Gemini 300 MHz on Bruker Avance III using tetramethylsilane as an internal standard (chemical shifts in δ scale), while ^13^C NMR spectra were run at 75 MHz. Elemental analyses were carried out at the Microanalytical Unit, Faculty of Science, Ain Shams University, using a Perkin-Elmer 2400 CHN elemental analyzer (Waltham, MA), and satisfactory analytical data (± 0.4) were obtained for all compounds. The homogeneity of the synthesized compounds was controlled by thin layer chromatography (TLC), using aluminum sheet silica gel F_254_ (Merck).

***2-(3-methoxy-3-oxo-1-(thiophen-2-yl)prop-1-en-2-yl)benzoic acid 1a***: A mixture of dimethyl homophthalate (2 g, 10 mmol) and thiophene-2-carbaldehyde (0.9 mL, 10 mmol) was stirred at 60 °C in dry methanol (20 mL) in the presence of sodium methoxide (0.5 g Na in 50 mL MeOH). The whole mixture was stirred for 5 h (TLC). The solid that precipitated was filtered off, dried, and then dissolved in cold water and acidified with cold dilute hydrochloric acid. The deposited solid was filtered off, washed several times with cold water, dried, and then recrystallized from benzene to afford **1** as buff crystals; mp 174–176 °C [Lit, mp 173–174 °C]^53^, yield 85%. ^1^H-NMR (400 MHz, DMSO) δ (ppm): 12.69 (br s, 1H, COOH, exchangeable with D_2_O), 8.12 (d, *J* = 7.6 Hz, 1H), 7.96 (s, 1H), 7.61 (dt, *J* = 14, 7.2 Hz, 2 H), 7.46 (d, *J* = 4.8 Hz, 1H), 7.34 (d, *J* = 2.8 Hz, 1H), 7.30 (d, *J* = 7.2 Hz, 1H), 6.99–6.97 (m, 1H), 3.64 (s, 3 H). ^13^C-NMR (100 MHz, DMSO) δ 167.5, 167.2, 138.5, 137.05, 134.2, 133.4, 132.2, 131.5 (2), 131.4, 131.1, 130.4, 129.4, 127.4, 52.2. Anal. calcd. for C_15_H_12_O_4_S (288.32): C, 62.49; H, 4.20; S, 11.12. Found: C, 62.44; H, 4.37; S, 11.21.

***(E)−2-(1-carboxy-2-(thiophen-2-yl)vinyl)benzoic acid 2***: The half ester **1a** (2.8 g, 10 mmol) was heated under reflux with (10%) sodium hydroxide (30 mL) for 2 h (TLC). The reaction mixture was allowed to cool and then acidified with cold dilute hydrochloric acid. The deposited solid was filtered off, washed several times with cold water, dried, and then recrystallized from ethanol to give **2** as pale yellow crystals; mp 218–220 °C [Lit, mp 215 °C]^53^, yield 76%. IR (υ/cm^−1^): br 3088 − 2528 (-OH), 1684 (C = O). Anal. calcd. for C_14_H_10_O_4_S (274.29): C, 61.31; H, 3.67; S, 11.69. Found: C, 61.28; H, 3.78; S, 11.60.

***(E)−4-(thiophen-2-ylmethylene)isochromane-1***,***3-dione 3***: A solution of dibasic derivative **2** (2.7 g, 10 mmol) in acetic anhydride (15 mL) was refluxed on water-bath for 2 h. The precipitated solid was filtered off, washed with light petroleum ether (b.p. 60–80 °C) then recrystallized from dioxane to give **3** as orange crystals; mp 158–160 °C, yield 73%. IR (υ/cm^−1^): 3101, 3086 (C-H_aromatic_), 1768, 1726 (C = O_vibrational coupling_). ^1^H-NMR (300 MHz, DMSO-*d*_6_) δ (ppm): 7.33 (t, 1H, Ar-H), 7.54 (t, 1H, Ar-H), 7.83 (t, 1H, Ar-H), 8.07–8.13 (m, 3 H, Ar-H), 8.26 (d, 1H, Ar-H, *J* = 8.1 Hz), 8.74 (s, 1H_olefinic_).^13^C-NMR (75 MHz, DMSO-*d*_6_) δ (ppm): 112.2, 119.9, 120.3, 122.7, 127.8, 128.2, 129.8, 135.2, 136.7, 137.3, 138.3, 139.3, 142.8, 160.2, 160.8. Anal. calcd. for C_14_H_8_O_3_S (256.28): C, 65.61; H, 3.15; S, 12.51. Found: C, 65.49; H, 3.35; S, 12.60.

***General procedure for synthesis of compounds (5a-f)***

A mixture of the anhydride derivative **3** (2.5 g, 10 mmol) and aromatic amines **4a-f** (20 mmol) in glacial AcOH (30 mL) was heated at 100 °C for 6–8 h. The obtained solid while heating and/or after concentration was filtered off, dried and then recrystallized from suitable solvents to give the corresponding compounds **5a-f**, respectively.

***(E)−4-(thiophen-2-ylmethylene)−2-(p-tolyl)isoquinoline-1***,***3(2 H***,***4 H)-dione (5a)***: Recrystallized from EtOH as green crystals; mp 260–262 °C, yield 87%. IR (υ/cm^−1^): 3100, 3063 (CH_aromatic_), 2917, 2853 (CH_aliphatic_), 1698 (C = O), 1651 (C = C). ^1^H-NMR (300 MHz, DMSO-*d*_6_) δ (ppm): 2.39 (s, 3 H, CH_3_), 7.16–7.32 (m, 5 H, Ar-H), 7.55 (t, 1H, Ar-H), 7.81 (t, 1H, Ar-H), 7.96–814 (m, 3 H, Ar-H), 8.33 (d, 1H, Ar-H, *J* = 8.1 Hz), 8.69 (s, 1H_olefinic_). ^13^C-NMR (75 MHz, DMSO-*d*_6_) δ (ppm): 20.8, 116.3, 120.5, 122.7, 123.8, 127.6, 128 (4), 129.4, 131.3, 133 (2), 135.2, 137 (3), 142.2, 163.5, 164. Anal. calcd. for C_21_H_15_NO_2_S (345.42): C, 73.02; H, 4.38; N, 4.06 S, 9.28. Found: C, 73.87; H, 4.29; N, 4.09; S, 9.08.

***(E)−2-(4-methoxyphenyl)−4-(thiophen-2-ylmethylene)isoquinoline-1***,***3(2 H***,***4 H)-dione (5b)***: Recrystallized from dioxane to give **5b** as pale green crystals; mp 282–284 °C, yield 79%. IR (υ/cm^−1^): 3090, 3061 (CH _aromatic_), 2948, 2831 (CH _aliphatic_), 1691 (C = O), 1647 (C = C). ^1^H-NMR (300 MHz, DMSO-*d*_6_) δ (ppm): 3.82 (s, 3 H, OCH_3_), 7.03–7.30 (m, 5 H, Ar-H), 7.55 (t, 1H, Ar-H), 7.81 (t, 1H, Ar-H), 7.97–8.15 (m, 3 H, Ar-H), 8.33 (d, 1H, Ar-H, *J* = 7.8 Hz), 8.70 (s, 1H_olefinic_). Anal. calcd. for C_21_H_15_NO_3_S (361.42): C, 69.79; H, 4.18; N, 3.88; S, 8.87. Found: C, 69.61; H, 4.22; N, 3.95; S, 8.74.

***(E)−2-(4-nitrophenyl)−4-(thiophen-2-ylmethylene)isoquinoline-1***,***3(2 H***,***4 H)-dione (5c)***: Recrystallized from dioxane as green crystals; mp > 300 °C, yield 69%. IR (υ/cm^−1^): 3093, 3074 (C-H_aromatic_), 1695 (C = O), 1649 (C = C). ^1^H-NMR (300 MHz, DMSO-*d*_6_) δ (ppm): 7.30 (d, 1H, Ar-H), 7.55–7.71 (m, 3 H, Ar-H), 7.83 (t, 1H, Ar-H), 7.98–8.16 (m, 3 H, Ar-H), 8.36–8.39 (m, 3 H, Ar-H), 8.77 (s, 1H_olefinic_). Anal. calcd. for C_20_H_12_N_2_O_4_S (376.39): C, 63.82; H, 3.21; N, 7.44; S, 8.52. Found: C, 63.73; H, 3.08; N, 7.52; S, 8.35.

***(E)−4-(1***,***3-dioxo-4-(thiophen-2-ylmethylene)−3***,***4-dihydroisoquinolin-2(1H)-yl)benzoic acid (5d)***: Recrystallized from dioxane as yellow crystals; mp > 300 °C, yield 71%. IR (υ/cm^−1^): br 3310 (OH), 3091, 3073 (C-H_aromatic_), 1722, 1690 (C = O). ^1^H-NMR (300 MHz, DMSO-*d*_6_) δ (ppm): 7.28–7.31 (m, 1H, Ar-H), 7.47–7.59 (m, 3 H, Ar-H), 7.83 (t, 1H, Ar-H), 7.98–8.16 (m, 5 H, Ar-H), 8.36 (d 1H, Ar-H, *J* = 8.4 Hz), 8.74 (s, 1H_olefinic_). ^13^C-NMR (75 MHz, DMSO-*d*_6_) δ (ppm): 116.1, 122.8, 123.6, 127(2), 128.5, 129(2), 130(2), 134.1, 135.3, 137(2), 140.2, 142.4, 163.4, 163.8, 166.9. Anal. calcd. for C_21_H_13_NO_4_S (375.40): C, 67.19; H, 3.49; N, 3.73; S, 8.54. Found: C, 67.11; H, 3.70; N, 3.92; S, 8.29.

***(E)−4-(1***,***3-dioxo-4-(thiophen-2-ylmethylene)−3***,***4-dihydroisoquinolin-2(1H)yl)benzene-sulfon-amide (5e)***: Recrystallized from dioxane as dark green crystals; mp > 300 °C, yield 65%. IR (υ/cm^−1^): br. 3228 (NH_2_), 3095 (C-H _aromatic_), 1686 (C = O), 1642 (C = C). ^1^H-NMR (300 MHz, DMSO-*d*_6_) δ (ppm): 7.29 (t, 1H, Ar-H), 7.49–7.57 (m, 3 H, Ar-H, + 2 H, NH_2_), 7.82 (t, 1H, Ar-H), 7.85–8.16 (m, 5 H, Ar-H), 8.35 (d, 1H, Ar-H, *J* = 8.1 Hz), 8.73 (s, 1H_olefinic_). ^13^C-NMR (75 MHz, DMSO-*d*_6_) δ (ppm): 116.1, 122.8, 123.6, 126.5, 127(2), 128.6, 130, 134.1, 137(3), 139.1, 142.5, 143.8, 163.2, 163.8. Anal. calcd. for C_20_H_14_N_2_O_4_S_2_ (410.46): C, 58.52; H, 3.44; N, 6.82; S, 15.62. Found: C, 58.36; H, 3.39; N, 6.96; S, 15.73.

***(E)−2-(1***,***5-dimethyl-3-oxo-2-phenyl-2***,***3-dihydro-1H-pyrazol-4-yl)−4-(thiophen-2-ylmethy-lene)isoquinoline-1***,***3(2 H***,***4 H)-dione (5f)***: Recrystallized from benzene as yellow crystals: mp 270–272 °C, yield 67%. IR (υ/cm^−1^): 3067 (C-H _aromatic_), 2967, 2930 (C-H _aliphatic_), 1699, 1663 (C = O). ^1^H-NMR (300 MHz, DMSO-*d*_6_) δ (ppm): 2.16 (s, 3 H, CH_3_), 3.23 (s, 3 H, N-CH_3_), 7.29–7.59 (m, 7 H, Ar-H), 7.82 (t, 1H, Ar-H), 8.01–8.16 (m, 3 H, Ar-H), 8.33 (d, 1H, Ar-H, *J* = 8.4 Hz), 8.72 (s, 1H_olefinic_). ^13^C-NMR (75 MHz, DMSO-*d*_6_) δ (ppm): 10.6, 35.6, 115.9, 122.9, 123.3, 124.1, 126.8, 127.8, 128(2), 129.3, 134(2), 134.4, 137.8, 138, 142.6, 154.3, 161.1, 163.2. Anal. calcd. for C_25_H_19_N_3_O_3_S (441.51): C, 68.01; H, 4.34; N, 9.52; S, 7.26. Found: C, 68.26; H, 4.44; N, 9.37; S, 7.37.

***(E)−4-(thiophen-2-ylmethylene)isoquinoline-1***,***3(2 H***,***4 H)-dione (6)***: A mixture of **3** (2.5 g, 10 mmol) and ammonium acetate (10 g) was fused in an oil bath at 170 °C for 30 min. The deposited solid on hot was filtered off, washed with water for several times, dried and then recrystallized from EtOH to give **6** as yellow crystals; mp 258–260 °C, yield 68%. IR (υ/cm^−1^): 3172 (NH), 3100, 3062 (C-H _aromatic_), 1705, 1670 (C = O). ^1^H-NMR (300 MHz, DMSO-*d*_6_) δ (ppm): 7.27–7.73 (m, 3 H, Ar-H), 8.00-8.25 (m, 4 H, Ar-H), 8.58 (s, 1H_olefinic_), 11.52 (br s, 1H, NH, exchangeable with D_2_O). ^13^C-NMR (75 MHz, DMSO-*d*_6_) δ (ppm): 116.1, 122.7, 123.6, 127(3), 133.6, 135.9, 136.5, 137(2), 141.9, 163.8, 164.5. Anal. calcd. for C_14_H_9_NO_2_S (255.29): C, 65.87; H, 3.55; N, 5.49; S, 12.56. Found: C, 65.95; H, 3.39; N, 5.74; S, 12.44.

***N-acetyl-5-oxo-1-(thiophen-2-yl)isochromeno[3***,***4-c]pyrazole-3(5 H)-carboxamide (7)***: A mixture of compound **3** (2.5 g, 10 mmol) and semicarbaide hydrochloride (1 g, 10 mmol) in glacial acetic acid (35 mL) in the presence of fused sodium acetate (1.5 g) was heated under reflux for 6 h. After cooling, the reaction mixture was poured into cold water. The deposited solid was filtered off, dried, and recrystallized from dioxane to give **7** as green crystals; mp 238–240 °C, yield 65%. IR (υ/cm^−1^): 3189 (NH), 3076, 3012 (C-H _aromatic_), 2809 (C-H _aliphatic_), 1717, 1672 (C = O). ^1^H-NMR (300 MHz, DMSO-*d*_6_) δ (ppm): 2.07 (s, 3 H, CH_3_), 7.32 (t, 1H, Ar-H), 7.56 (t, 1H, Ar-H), 7.81 (t, 1H, ArH), 8.07–8.15 (m, 3 H, Ar-H), 8.31 (d, 1H, Ar-H, *J* = 7.5 Hz), 8.73 (s, 1H_olefinic_), 10.57 (s, 1H, NH, exchangeable with D_2_O). ^13^C-NMR (75 MHz, DMSO-*d*_6_) δ (ppm): 20.5, 115.4, 123, 127.9, 128(2), 134(2), 137.5, 138(2), 143, 161.5, 161.8, 168.3. Anal. calcd. for C_17_H_11_N_3_O_4_S (353.35): C, 57.79; H, 3.14; N, 11.89; S, 9.07. Found: C, 57.90; H, 3.06; N, 11.96; S, 9.11.

***(E)−6-(thiophen-2-ylmethylene)−2-thioxo-2,6-dihydro-[1,2,4]triazolo[5,1-a]isoquinolin-5(3 H)-one (8)***: A mixture of compound 3 (2.5 g, 10 mmol) and thiosemicarbazide (0.9 g, 10 mmol) in glacial acetic acid (30 mL) was refluxed for 3 h. The precipitated solid while heating was filtered off, washed with ethanol, dried and then crystallized from EtOH to give **8** as orange crystals; mp 248–250 °C, yield 84%. IR (υ/cm^−1^): 3361, 3307, 3201 (NH), 1701 (C = O), 1654 (C = C). ^1^H-NMR (300 MHz, DMSO-*d*_6_) δ (ppm): 7.32 (t, 1H, Ar-H), 7.55 (t, 1H, Ar-H), 7.77–8.07 (m, 3 H, Ar-H), 8.14 (d, 1H, Ar-H, J = 7.8 Hz), 8.30 (d, 1H, Ar-H, J = 8.7 Hz), 8.69 (s, 1Holefinic), 9.66 (s, 1H, NH, exchangeable with D2O). ^13^C-NMR (75 MHz, DMSO-*d*_6_) δ (ppm): 116.5, 122.8, 123.8, 127(2), 128.7, 134.1, 135.2, 137.7, 138, 142,7, 161.9, 162.2, 181.7. Anal. calcd. for C_15_H_9_N_3_OS_2_ (311.38): C, 57.86; H, 2.91; N, 13.50; S, 20.59. Found: C, 57.71; H, 2.84; N, 13.40; S, 20.72.

***(E)-N-(1***,***3-dioxo-4-(thiophen-2-ylmethylene)−3***,***4-dihydroisoquinolin-2(1H)-yl)−4-methyl-benzenesulfonamide (9)***: A mixture of compound 3 (2.5 g, 10 mmol) and *p*-toluene sulfonohydrazide (1.85 g, 10 mmol) in glacial acetic acid (30 mL) was heated under reflux for 12 h. The excess solvent was removed under reduced pressure, and the deposited solid was collected by filtration, dried and recrystallized from EtOH to give **9** as green crystals; mp 230–232 °C, yield 66%. IR (υ/cm^−1^): 3218 (NH), 3094, 3079 (C-Haromatic), 2958, 2877 (C-Haliphatic), 1711, 1672 (C = O). ^1^H-NMR (300 MHz, DMSO-*d*_6_) δ (ppm): 2.5(s, 3 H, CH3), 7.31(d, 3 H, Ar-H), 7.55 (t, 1H, Ar-H), 7.70 (d, 1H, Ar-H, J = 6.9 Hz), 7.80 (t, 2 H, Ar-H), 8.02–8.09 (m, 3 H, Ar-H), 8.27 (d, 1H, Ar-H, J = 7.8), 8.66 (s, 1Holefinic), 10.65 (s, 1H, NH, exchangeable with D2O). ^13^C-NMR (75 MHz, DMSO-*d*_6_) δ (ppm): 21, 115.4, 122.9, 127(2), 128(2), 129.8, 134(2), 137(2) 138(2), 142.7, 143.5, 161(2). Anal. calcd. for C_21_H_16_N_2_O_4_S_2_ (424.49): C, 59.42; H, 3.80; N, 6.60; S, 15.11. Found: C, 59.34; H, 3.94; N, 6.35; S, 15.2.

### Biological assay

A laboratory colony of *Culex pipiens* was successfully maintained for approximately 40 generations within the Entomology Department, Faculty of Science, Ain Shams University, adhering to standard operating procedures. The insectary environment was meticulously controlled, with a temperature of 27 ± 2 °C, a 12:12 h light-dark cycle, and 75% relative humidity. Following hatching, pupae were transferred to wooden cages (25 × 30 × 25 cm). Larvae were nourished with TetraMin, a commercially available fish food. Female mosquitoes were provided with blood meals from pigeons, while adult mosquitoes received a 10% sucrose solution daily for sustenance^61–64,72,73^.

#### Larvicidal bioassay

Larvicidal bioassays were conducted according to the World Health Organization (WHO) recommended protocol^74^. To evaluate the insecticidal efficacy of the test compounds, twenty third-instar *Culex pipiens* larvae were exposed to a series of concentrations: 11.25, 22.5, 45, 67.5, and 90 µg/mL. The test compounds were initially dissolved in dimethylformamide (DMF) and then diluted with distilled water to the final concentrations. Each treatment was tested in triplicate (*n* = 3). A positive control using chlorpyrifos was included to validate the bioassay protocol and serve as a benchmark for larvicidal activity. Chlorpyrifos was tested at concentrations of 10, 50, 100, 150, and 200 µg/mL, with three replicates per concentration. A negative control group, consisting of larvae exposed only to DMF diluted in water (without any test compound), was included and also replicated three times. The average mortality observed in this control group was used to correct the mortality in both the test compound groups and the positive control group using Abbott’s formula^65^. Larval mortality was assessed after 24 h of exposure and was defined as the absence of a response to gentle prodding^61–64,72–74^.

#### Data analysis

Larval mortality data for the tested compounds and the positive control chlorpyrifos, corrected using Abbott’s formula^65^, were subjected to statistical analysis using the LDP line program. Lethal concentration values (LC₅₀) were calculated along with their corresponding 95% confidence intervals. The Finney formula, Chi-square test, and coefficient of determination (r²) were applied to evaluate the accuracy and goodness-of-fit of the experimental data^75^. Additionally, the toxicity index (TI) of the test compounds against mosquito larvae was calculated using the Sun equation^76^.

### Molecular docking assessment

To explore the potential mode of action underlying the observed larvicidal activity of the synthesized compounds, molecular docking simulations were carried out using the Molecular Operating Environment (MOE) software (version 2024.06; https://www.chemcomp.com/en/index.htm). The two-dimensional (2D) structures of the tested compounds were generated using ChemDraw 20.0 and converted into three-dimensional (3D) structures in MOE. Protonation states were assigned, partial charges calculated, and initial energy minimization was performed to prepare the compounds for docking studies^61–64,73,77^.

#### Acetylcholinesterase (AChE) docking

Molecular docking of the test compounds against acetylcholinesterase (AChE) was performed using the crystal structure of an insecticide-resistant AChE mutant from *Anopheles gambiae*, co-crystallized with a difluoromethyl ketone inhibitor (PDB ID: 6ARY). The structure includes the ligand BT7 ((1 S)−2,2-difluoro-1-[1-(pentan-3-yl)−1 H-pyrazol-4-yl]ethan-1-ol), which was retained to define the active site. The protein was retrieved from the Protein Data Bank, and all non-essential water molecules and ligands were removed, except for BT7. Hydrogen atoms were added, and the structure was energy-minimized prior to docking. The receptor was kept rigid during simulations, while ligand flexibility was allowed. The binding site was defined around the co-crystallized ligand BT7.

To validate the docking protocol, BT7 was redocked into the enzyme’s active site using the same parameters. The resulting pose yielded an RMSD of 0.78 Å compared to the crystallographic conformation, confirming the accuracy and reliability of the docking procedure. Docking simulations for the test compounds were then carried out, generating 100 initial poses per ligand. The top 20 poses were ranked based on binding scores (London ΔG), and the most favorable pose was selected based on the lowest predicted binding energy (S-score), RMSD below 2 Å, and interaction similarity to BT7. Binding modes were further evaluated for key interactions, including hydrogen bonding, ionic contacts, and π-stacking.

As an additional reference, **chlorpyrifos**, a known AChE inhibitor, was docked into the same active site, and its binding mode was compared with those of the test compounds and the redocked BT7 ligand.

#### Nicotinic acetylcholine receptor (nAChR) docking

For the molecular docking of compounds against the nicotinic acetylcholine receptor (nAChR), which lacks an experimentally resolved 3D structure in the PDB, a homology model was constructed using the Swiss-Model server. The sequence for the β2 subunit of the nAChR was retrieved from UniProt (ID: B0XBN4) and modeled using the Swiss-Model homology server, providing a reliable template for docking studies. The model was further validated by calculating its GMQE (Global Model Quality Estimation) score of 0.91 and the MolProbity score of 1.26, indicating the model’s quality. Alpha pockets within the protein structure were identified using the Site-Finder function implemented in MOE, and the identified pocket was used for docking the compounds. For the docking simulations, the docking grid was set on the predicted Alpha pocket. As a reference, thiamethoxam, a well-known nAChR agonist, was used to validate the docking results. The structural similarities between thiamethoxam and some of the tested compounds were observed in their common aromatic structures and nitrogen-containing heterocycles, which might contribute to their similar binding modes. The top docking poses were selected based on binding affinity (S-score), key interactions, and RMSD values.

### Molecular dynamics simulations

Molecular dynamics (MD) simulations were conducted to evaluate the dynamic behavior and binding stability of four compounds in complex with acetylcholinesterase (AChE; PDB ID: 6ARY): the precursor molecule 1a, the most potent thiophene–isoquinolinone derivative compound 6, the reference AChE inhibitor chlorpyrifos, and the redocked co-crystallized ligand BT7. All simulations were performed using the Desmond module (Schrödinger Suite, Schrödinger, LLC, New York, NY, USA) under identical conditions to ensure direct comparability.

The crystal structure of AChE (PDB ID: 6ARY), which represents an insecticide-resistant variant, was obtained from the Protein Data Bank. Initial protein–ligand complexes were prepared using the top-ranked docking poses from prior analyses, selected based on binding affinity and interaction profiles. Each complex (1a–AChE, 6–AChE, chlorpyrifos–AChE, and BT7–AChE) was solvated in an explicit TIP3P water box with a 10 Å buffer region and neutralized with Na⁺ and Cl⁻ ions to achieve a physiological salt concentration of 0.15 M NaCl. The OPLS4 force field was used to model all molecular interactions, and ligand parameters were generated using the LigPrep module^60,62^.

Prior to the production run, each system underwent energy minimization (2000 steps of Steepest Descent followed by Conjugate Gradient optimization). Production MD simulations were then carried out for 100 nanoseconds in the NPT ensemble, maintaining a temperature of 300 K (Nose–Hoover thermostat) and pressure of 1 atm (Martyna–Tobias–Klein barostat). Long-range electrostatics were computed using the Particle Mesh Ewald (PME) method, and short-range interactions were calculated using a 9.0 Å cutoff. The SHAKE algorithm was used to constrain bonds involving hydrogen atoms, enabling a 2-fs integration timestep^60,62,71^.

Trajectory analyses were conducted using the Simulation Interaction Diagram (SID) tool in Desmond. The main descriptors emphasized include Root Mean Square Deviation (RMSD) for assessing global stability, Root Mean Square Fluctuation (RMSF) for evaluating local residue flexibility—especially in the binding site—and ligand–protein contact histograms and interaction maps to quantify the frequency and persistence of hydrogen bonds, hydrophobic interactions, and π–π stacking. Additional metrics such as radius of gyration (Rg), solvent-accessible surface area (SASA), and total system energy were also evaluated and are provided in the Supplementary Information **(Supplementary Data)**. Together, these analyses offer a detailed comparative assessment of the binding behavior and dynamic stability of compound 1a, compound 6, chlorpyrifos and BT7 within the AChE active site.

## Conclusion

In this study, we successfully designed, synthesized, and evaluated a novel series of thiophene–isoquinolinone hybrids for their larvicidal activity against *Culex pipiens*. The compounds demonstrated potent biological activity, with derivatives 6, 5f, and 7, along with the thiophene-based half-ester precursor 1a, exhibiting significantly greater toxicity than the commercial insecticide chlorpyrifos. Notably, compound 1a showed the highest larvicidal potency (LC₅₀ = 0.004 µg/mL), underscoring its potential as a lead scaffold for further development.

Mechanistic studies using molecular docking and molecular dynamics simulations revealed strong and specific interactions of the most active compounds with both acetylcholinesterase (AChE) and nicotinic acetylcholine receptors (nAChRs), suggesting a dual-target neurotoxic mode of action. These observations were further supported by DFT calculations, which confirmed the electronic favorability and chemical reactivity of the active molecules. Structure–activity relationship (SAR) analysis emphasized the importance of preserving electrophilic and conjugated features—particularly the α,β-unsaturated ketone motif—for optimizing larvicidal efficacy.

The synthetic flexibility and structural diversity of the thiophene–isoquinolinone framework offer considerable potential to address insecticide resistance through rational design and molecular optimization. Collectively, these findings highlight this chemotype as a promising candidate for the development of next-generation larvicides targeting critical neurophysiological pathways in mosquito vectors. Future work should focus on in vitro validation, ecological safety profiling, and advanced pharmacological studies to facilitate potential application in integrated mosquito control strategies.

## Supplementary Information

Below is the link to the electronic supplementary material.

Supplementary Material 1

## Data Availability

All data generated or analyzed during this study are included in this published article and its supplementary information files.
